# Virtual screening approach to identifying influenza virus neuraminidase inhibitors using molecular docking combined with machine-learning-based scoring function

**DOI:** 10.18632/oncotarget.20915

**Published:** 2017-09-15

**Authors:** Li Zhang, Hai-Xin Ai, Shi-Meng Li, Meng-Yuan Qi, Jian Zhao, Qi Zhao, Hong-Sheng Liu

**Affiliations:** ^1^ School of Life Science, Liaoning University, Shenyang 110036, China; ^2^ Research Center for Computer Simulating and Information Processing of Bio-macromolecules of Liaoning Province, Shenyang 110036, China; ^3^ Engineering Laboratory for Molecular Simulation and Designing of Drug Molecules of Liaoning, Shenyang 110036, China; ^4^ School of Mathematics, Liaoning University, Shenyang 110036, China

**Keywords:** influenza virus, neuraminidase inhibitor, virtual screening, machine learning, scoring function

## Abstract

In recent years, an epidemic of the highly pathogenic avian influenza H7N9 virus has persisted in China, with a high mortality rate. To develop novel anti-influenza therapies, we have constructed a machine-learning-based scoring function (RF-NA-Score) for the effective virtual screening of lead compounds targeting the viral neuraminidase (NA) protein. RF-NA-Score is more accurate than RF-Score, with a root-mean-square error of 1.46, Pearson’s correlation coefficient of 0.707, and Spearman’s rank correlation coefficient of 0.707 in a 5-fold cross-validation study. The performance of RF-NA-Score in a docking-based virtual screening of NA inhibitors was evaluated with a dataset containing 281 NA inhibitors and 322 noninhibitors. Compared with other docking–rescoring virtual screening strategies, rescoring with RF-NA-Score significantly improved the efficiency of virtual screening, and a strategy that averaged the scores given by RF-NA-Score, based on the binding conformations predicted with AutoDock, AutoDock Vina, and LeDock, was shown to be the best strategy. This strategy was then applied to the virtual screening of NA inhibitors in the SPECS database. The 100 selected compounds were tested in an *in vitro* H7N9 NA inhibition assay, and two compounds with novel scaffolds showed moderate inhibitory activities. These results indicate that RF-NA-Score improves the efficiency of virtual screening for NA inhibitors, and can be used successfully to identify new NA inhibitor scaffolds. Scoring functions specific for other drug targets could also be established with the same method.

## INTRODUCTION

Since the first report of human infection with the avian influenza H7N9 virus in China in March 2013, this new viral subtype has caused sustained annual epidemic in subsequent years [1, 2]. The newly issued monthly World Health Organization (WHO) risk assessment summary at the human–animal interface [3] has reported that, as of June 2017, avian influenza H7N9 has caused 1533 laboratory-confirmed human infections, including at least 592 deaths. Recent studies have shown that the pandemic potential of avian influenza H7N9 is great [4, 5]. The emergence of new types of influenza viruses and the spread of drug-resistant strains [6] make the influenza virus a serious public-health threat.

Neuraminidase (NA) is an important surface glycoprotein of the influenza viruses. The main role of NA is to remove the sialic acid groups from glycoproteins at the surfaces of host cells, resulting in the release of virion progeny from infected cells. NA also allows the virus to move through the mucus layer of the respiratory tract in the early stage of infection, which is important if the virus is to reach and infect epithelial cells [7, 8]. NA also prevents the formation of viral aggregates at the surfaces of host cells, enhancing viral infectivity [9]. The amino acid residues in the active site of NA are highly conserved among all natural strains of influenza virus infections [10]. These features of NA make it an attractive target for the control and treatment of influenza virus [11]. Several NA inhibitors that target the active site of NA have been developed and shown to be effective in clinical trials [12-15]. Two NA inhibitors, oseltamivir (Tamiflu®) and zanamivir (Relenza®), have been approved by the Food and Drug Administration (FDA) in the USA for the treatment and prevention of influenza viral infection. However, the emergence and wide-ranging spread of oseltamivir-resistant strains [6, 16], the limited use of zanamivir because its oral bioavailability is poor [17], and the emergence of new and more aggressive strains, such as avian H5N1 and H7N9 [1], have amplified the need for the development of new and more-effective antiviral compounds.

Virtual screening based on molecular docking is becoming a powerful tool in identifying lead compounds [18, 19], and a series of potential NA inhibitors has been identified with this method [20-22]. Current NA inhibitor virtual screening studies usually use the score (binding affinity value) given by scoring functions integrated into molecular docking software to rank the compounds. Therefore, this score is the main basis for the selection of potential inhibitors. These scoring functions are designed to recognize correct binding conformations in the docking process, but perform poorly in ranking the compounds correctly in the order of their activity against a target. Cheng et al. examined the abilities of 16 popular scoring functions to predict binding affinities using a dataset containing 195 protein–ligand complexes and their experimental binding affinities, and showed that the best scoring function achieved a Pearson’s correlation coefficient of 0.644 [23]. The low accuracy of the scoring function is a major factor limiting the efficiency of molecular-docking-based virtual screening [24].

In recent years, machine-learning methods have been successfully used in many aspects of pharmaceutical research, such as drug-target interactions prediction [25, 26], synergistic drug combinations prediction [27, 28], and drug toxicity prediction [29]. In the scope of virtual screening, new scoring functions based on modern machine-learning regression models have been introduced, and have been shown to outperform a wide range of classical scoring functions [30]. Khamis et al. [31] comprehensively evaluated 12 machine-learning-based scoring functions and 20 classical scoring functions. Their results showed that the machine-learning-based scoring functions were a substantial improvement on classical scoring functions in both scoring power (binding affinity prediction) and ranking power (relative ranking prediction). Ashtawy and Mahatrapa [32] reported that machine-learning scoring functions using the random forests algorithm or boosted regression trees were most frequently associated with the best performance. With the accumulation of protein–ligand complex structural data in public databases, it is possible to design scoring functions for the more-efficient prediction of the binding affinity of specific proteins or protein families.

In this study, we developed an NA-specific scoring function using the random forests algorithm for the efficient virtual screening of NA inhibitors. The efficiency of various virtual screening strategies combining different docking software and scoring functions was then evaluated on a test dataset containing 281 NA inhibitors and 322 noninhibitors. The best strategy was used to virtually screen for NA inhibitors in the SPECS database.

## RESULTS

### Performance of RF-NA-Score

Using 36 RF-Score features, 11 Vina features, and the experimental binding affinity values of the training set complexes (67 NA–ligand complexes from the PDBbind database version 2016) as input data, a scoring function specific for *Influenza A virus* NA, designated RF-NA-Score, was trained with the method proposed by Ballester and Mitchell [33-35]. The performance of RF-NA-Score was rigorously validated with 5-fold cross-validation (5-CV) and leave-one-out cross-validation (LOOCV) methods. The performance measures are presented in Table 1. For comparison, RF-Score was also retrained on the refined set of the latest version of the PDBbind database (version 2016), which contains more complexes and should result in a more robust scoring function. The performance of RF-Score in predicting the binding affinities of the 67 NA–ligand complexes is also shown in Table 1.

**Table 1:** Performance measures of RF-NA-Score and RF-Score for 67 NA–ligand complexes, measured with the root-mean-square error (RMSE), Pearson’s correlation coefficient (Rp), and Spearman’s rank correlation coefficient (Rs) for the predicted and measured binding affinities

Method	Number of test complexes	RMSE	Rp	Rs
5-CV fold1	13	1.47	0.846	0.841
5-CV fold2	13	1.86	0.740	0.714
5-CV fold3	13	1.51	0.630	0.758
5-CV fold4	14	1.57	0.577	0.565
5-CV fold5	14	0.88	0.744	0.657
5-CV average	13	1.46	0.707	0.707
70% resampling average	20	1.50	0.712	0.717
LOOCV	67	1.48	0.722	0.711
RF-NA-Score	67	1.63	0.670	0.593

As shown in Table 1, RF-NA-Score achieved root-mean-square errors (RMSEs) of 1.46, 1.48, and 1.50 on 5-CV, LOOCV, and resampling test, respectively, which are obviously smaller than the value of 1.63 achieved with RF-Score, indicating that the difference between the measured binding affinities and those predicted by RF-NA-Score was smaller than that of RF-Score. On two other performance measures, Pearson’s correlation coefficient (Rp) and Spearman’s rank correlation coefficient (Rs), RF-NA-Score was also better than RF-Score. This means that RF-NA-Score not only produces a higher linear correlation between the predicted and measured binding affinities, but also predicts a more accurate binding affinity rank for the ligands. These features should make virtual screening more efficient. The performance measures for RF-NA-Score and RF-Score suggest that RF-NA-Score is more accurate in predicting the binding affinities between ligands and NA, and could therefore be more efficient in the virtual screening of NA inhibitors.

### Validation of the accuracy of molecular docking software

**Figure 1:**
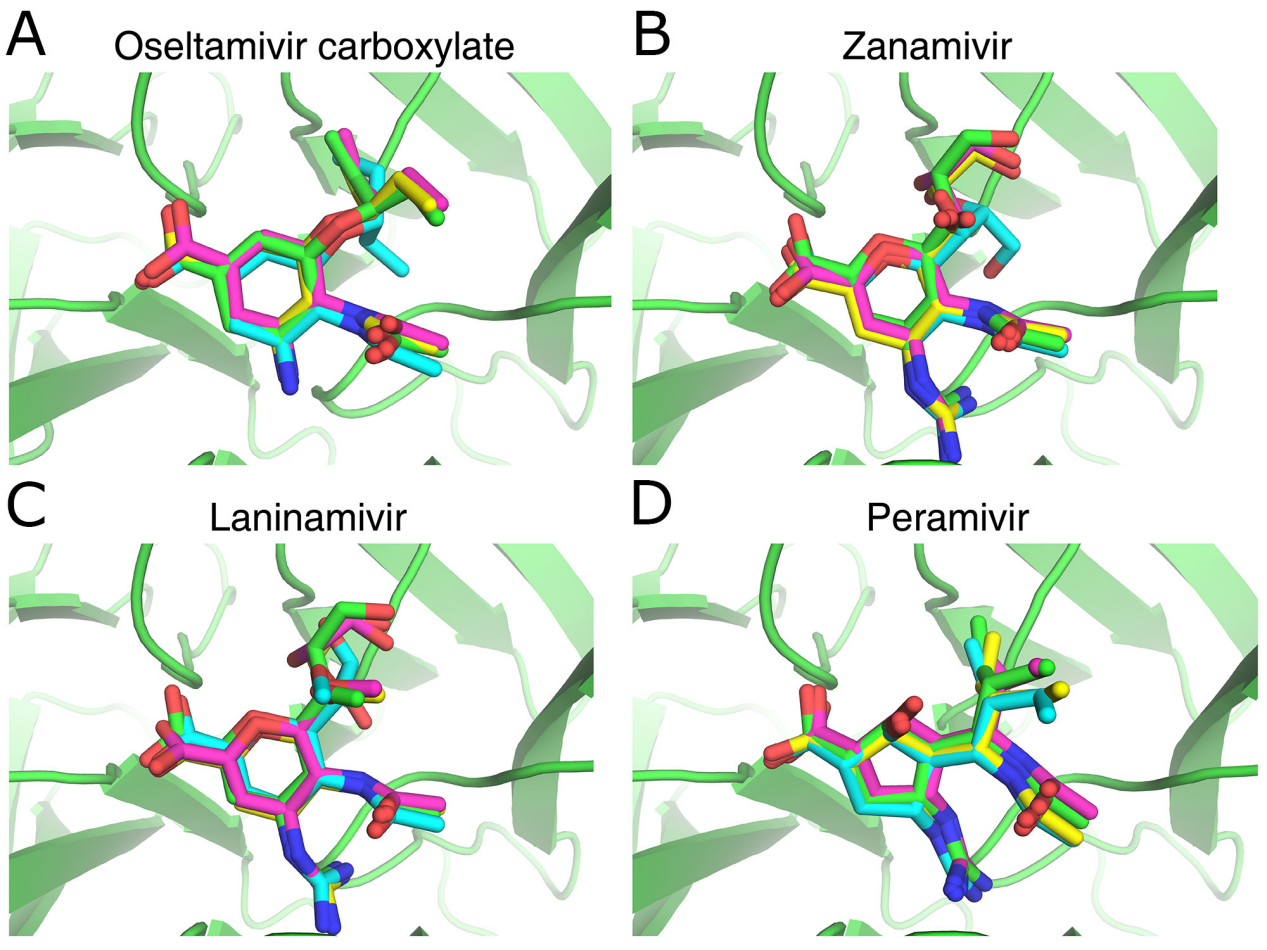
Comparison of the NA inhibitor binding conformations predicted with the docking softwaretools and their corresponding crystal structures **(A)** Oseltamivir carboxylate, PDB ID: 4MWQ; **(B)** zanamivir, PDB ID: 4MWR; **(C)** laninamivir, PDB ID: 4MWU; **(D)** peramivir, PDB ID: 4MWV. Small molecules with green, cyan, magenta, and yellow skeletons were derived from crystal structures and structures predicted with AutoDock, AutoDock Vina, and LeDock, respectively. The figure was prepared with PyMOL.

Because the accurate prediction of the complex structures combining NA and ligands is important for the use of scoring functions such as RF-NA-Score in virtual screening, we validated the accuracy of AutoDock, AutoDock Vina, and LeDock by comparing the predicted and experimentally determined complex structures of four NA inhibitors (oseltamivir carboxylate, zanamivir, laninamivir, and peramivir). Figure 1 shows the structures of NA complexed with oseltamivir carboxylate (Figure 1A), zanamivir (Figure 1B), laninamivir (Figure 1C), and peramivir (Figure 1D) predicted by AutoDock (cyan), AutoDock Vina (magenta), and LeDock (yellow), superimposed on their corresponding crystal structures (green). Table 2 shows the root-mean-square deviation (RMSD) between the predicted and experimentally determined binding conformations of the four inhibitors. It can be seen from Figure 1 that all the predicted binding conformations are very close to the crystal structures. The RMSD values (Table 2) are very low, with an average of around 0.76 Å and no values > 2 Å, especially for AutoDock Vina and LeDock, in which the RMSD values are all < 1 Å. These results indicate that all three molecular docking software tools are accurate in docking ligands to influenza NA.

**Table 2:** RMSD (in Å) between the predicted and experimentally determined binding conformations of four inhibitors

Software	Oseltamivir carboxylate	Zanamivir	Laninamivir	Peramivir
AutoDock	1.43	1.55	1.23	0.58
AutoDock Vina	0.41	0.61	0.65	0.43
LeDock	0.35	0.69	0.69	0.50

### Evaluation of RF-NA-Score in the virtual screening of NA inhibitors

With a comprehensive search of the literature and the bindingDB database (see details in Materials and Methods), we collected a ligand dataset containing 281 inhibitors and 322 noninhibitors of the group 2 NA enzymes of the influenza viruses. With this dataset, RF-NA-Score was evaluated for its effectiveness in virtually screening for influenza virus NA inhibitors using the strategy illustrated in Figure 1. Briefly, these ligands were docked to the crystal structure of H7N9 NA (PDB ID: 4MWJ) using the three docking software tools AutoDock, AutoDock Vina, and LeDock. The top-scoring binding complexes predicted by each software were then rescored with RF-Score and RF-NA-Score. The performances of the virtual screening strategies using the different docking software tools combined with different scoring methods were evaluated by comparing their score distributions of inhibitors and noninhibitors and their receiver operating characteristic (ROC) curves.

**Figure 2:**
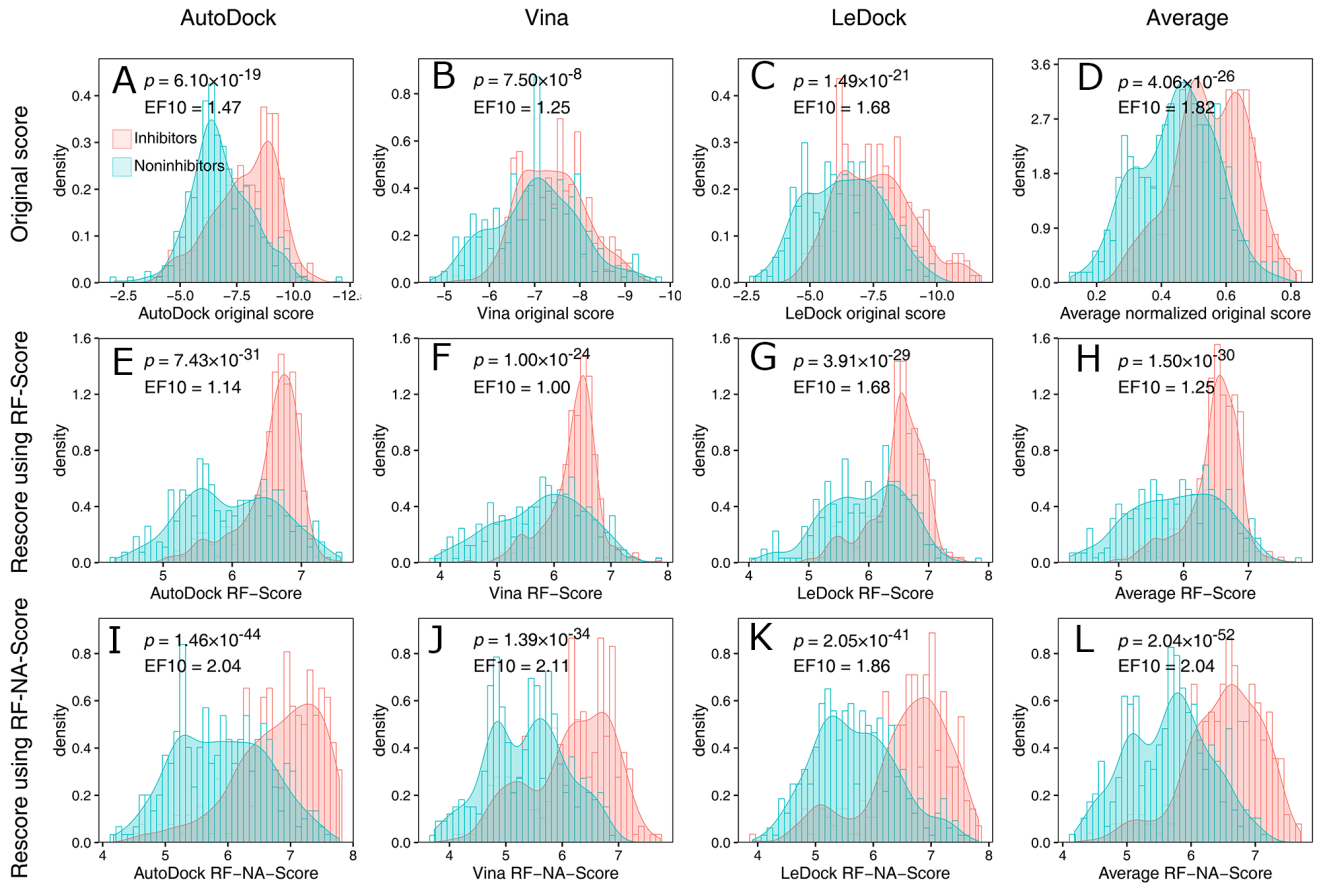
Score distributions of inhibitors (red) and noninhibitors (blue) provided by the docking software **(A, B, C, D)**, RFScore **(E, F, G, H)**, and RF-NA-Score **(I, J, K, L)** based on the top-scoring docking results of AutoDock (A, E, I), AutoDock Vina (B, F, J), and LeDock (C, G, K), and the average scores of these three software tools (D, H, L). To average the original scores, the scores were normalized to a range of 0–1 and then the average was calculated, because different scoring ranges are given by different docking software tools. The p values were calculated with Student’s t test. EF10 is the enrichment factor at 10%. The upper bound of EF10 is 2.15.

The score distributions of the inhibitors and noninhibitors are shown in Figure 2. Figure 2A-2C show the distributions of the original scores of the docking software tools. Although the peak values for the distributions of the inhibitors are greater than those of the noninhibitors for all three software tools, the distributions are strongly overlapping, which makes it difficult to distinguish inhibitors from noninhibitors. Rescoring with RF-Score increased the separation of the peak values and reduced the overlap (Figure 2E-2G), indicating that the effectiveness of virtual screening was improved by RF-Score. However, in the region with high scores (e.g., RF-Score > 7), the inhibitors and noninhibitors still seriously overlapped. This can be quantified by the enrichment factor at 10% (EF10) which reflects the power of the virtual screening method in the enrichment of inhibitors in top 10% of results. As shown in Figure 2E-2G, the values of EF10 are decreased (except LeDock) after rescoring with RF-Score. Figure 2I-2K show the distribution of the scores provided by RF-NA-Score. RF-NA-Score clearly outperforms RF-Score and the original scores. The separation of the peak values is significantly improved and the overlap between the inhibitors and noninhibitors is clearly smaller than those resulting from RF-Score and the original scores. There are also significantly more inhibitors in the region of high scores. The values of EF10 are significantly improved. Furthermore, the EF10 value of Vina (2.11) is close to the upper bound of 2.15. These results illustrate the effectiveness of RF-NA-Score in virtual screening.

We also computed an average score for each scoring method by averaging the scores of the three complex structures predicted with the different docking software tools. The distributions of the average scores are shown in Figure 2D, 2H, and 2L. Averaging the scores reduced the overlap of score distributions of inhibitors and noninhibitors for all three scoring methods. And the average RF-NA-Score displayed the best performance. The EF10 value of average RF-NA-Score is 2.04 which is higher than that of the other two average method (1.82 and 1.25) and slightly lower than that of vina combined with RF-NA-Score (2.11).

Student’s *t* test was used to evaluate the significance of the differences between the mean scores for the inhibitors and noninhibitors. The p value for the average RF-NA-Score strategy was 2.04 × 10^−52^, which was the lowest p value obtained for all strategies, and clearly suggests that the average RF-NA-Score outperformed the other strategies.

**Figure 3:**
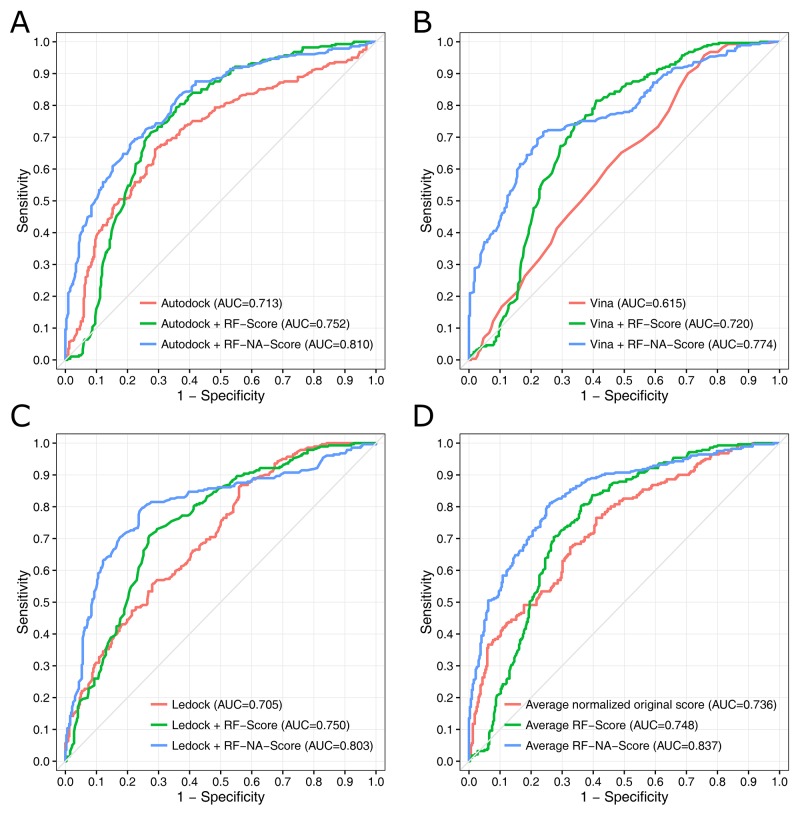
ROC curves for the virtual screening strategies using the docking software tools AutoDock **(A)**, AutoDock Vina **(B)**, and LeDock **(C)** combined with different scoring methods: original score (red), RF-Score (green), and RF-NA-Score (blue). Strategy using the average scores of the three docking software tools **(D)**.

The ROC curves and the areas under the ROC curves (AUCs) are presented in Figure 3. The ROC curve analysis is a well-recognized method of evaluating how good a model is at selecting known active molecules and discarding inactive molecules [36, 37]. The AUC values range from 0.5 (corresponding to a random model) to 1 (corresponding to an ideal model). In general, the greater the AUC, the more effective the virtual screening strategy is in discriminating active from inactive compounds. Comparing the AUC values of the different strategies clearly showed that RF-NA-Score outperformed the original score and RF-Score when combined with any of the three docking software tools. Figure 3 demonstrates that the best strategy is the average RF-NA-Score, which achieved an AUC value of 0.837. Overall, the results obtained from the ROC curve analysis are consistent with those obtained by comparing the scores distributions.

These results suggest that rescoring with RF-NA-Score significantly improves the efficiency of virtual screening for influenza virus NA inhibitors. Among these virtual screening strategies, the best strategy involved docking with AutoDock, AutoDock Vina, or LeDock, rescoring with RF-NA-Score, and then averaging the scores. This strategy was used in subsequent virtual screening.

### Screening the SPECS database

The best virtual screening strategy was used to screen candidate inhibitors of NA in a compound library containing 52,631 lead-like compounds (250 < molecular weight < 350, and logP < 3.5) in the SPECS database. After virtual screening, the 1000 compounds with the best average RF-NA-Score scores were clustered, and 100 compounds with wide chemical diversity were selected as candidate inhibitors.

**Figure 4:**
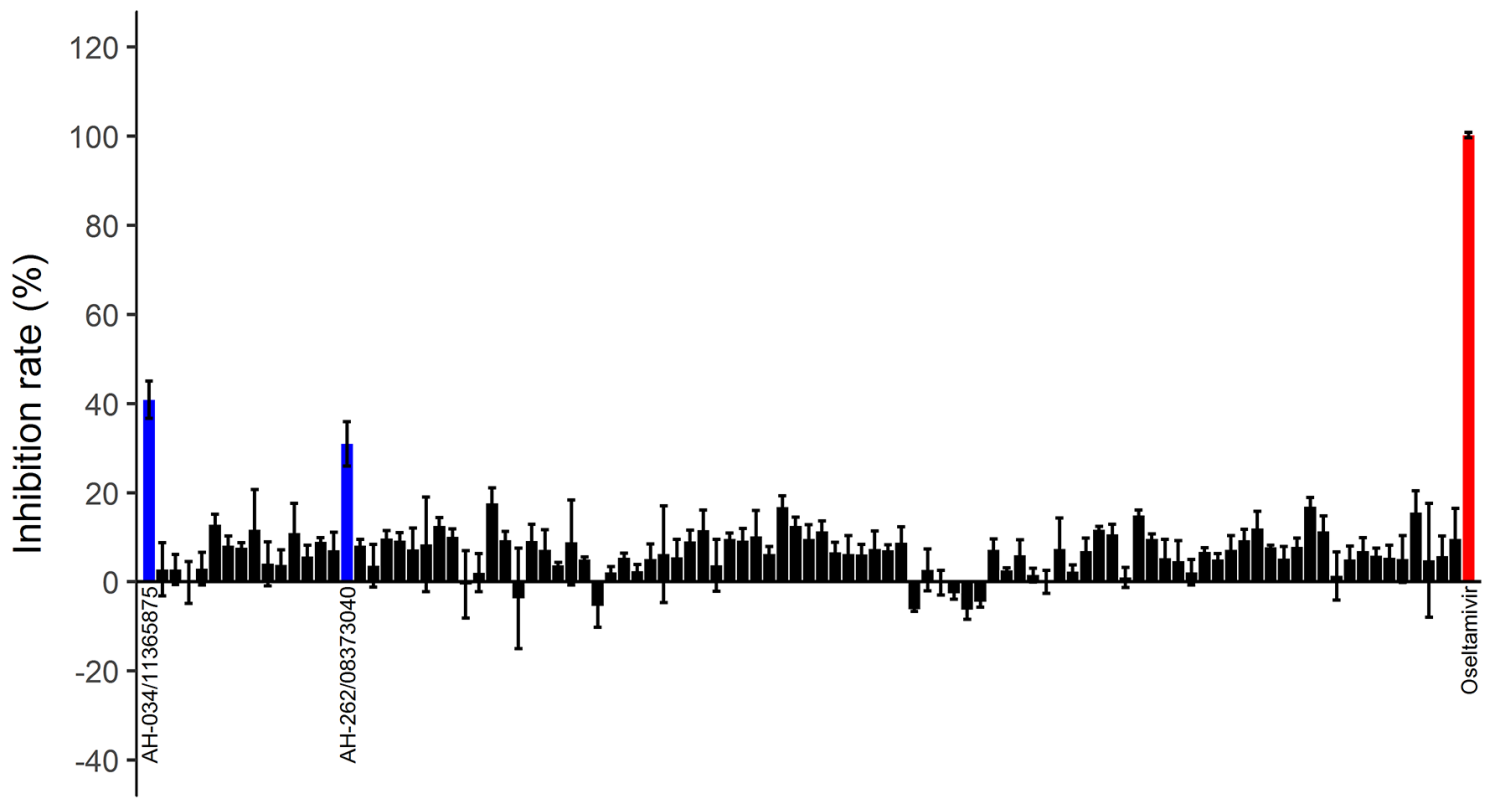
Inhibition rates (%) of 100 candidate inhibitors at concentrations of 100 μM Oseltamivir carboxylate was used as the positive control (red column). Two compounds (blue columns) inhibited NA activity by > 30%.

**Figure 5:**
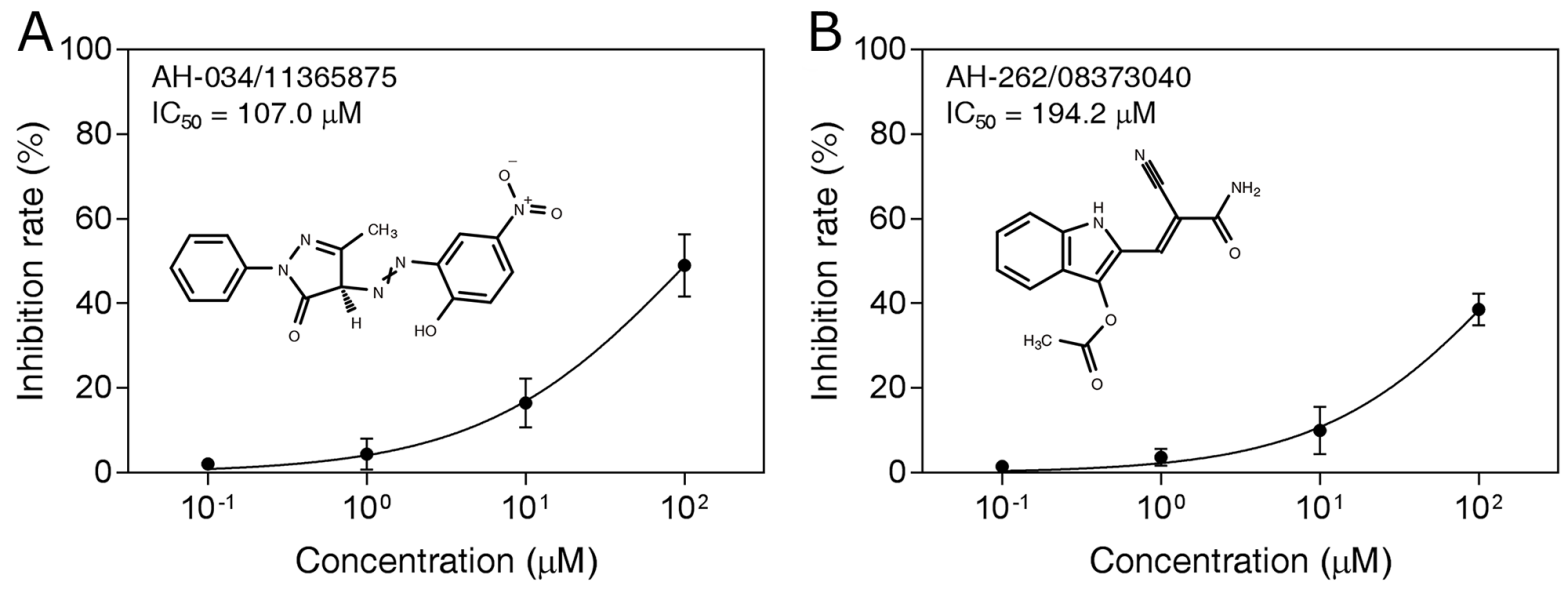
Dose-dependent inhibitory effects (IC50 values) of compounds AH-034/11365875 **(A)** and AH-262/08373040 **(B)**.

These compounds were tested in an *in vitro* H7N9 NA inhibition assay, using oseltamivir carboxylate as the positive control. Two of the compounds, AH-034/11365875 and AH-262/08373040, were found to be active at concentrations of 100 μM (Figure 4), with inhibition rates of 40.8% and 31.0%, respectively. The dose–response effects of these two compounds were then evaluated to determine the half-maximal inhibitory concentrations (IC_50_), which were 107.0 μM and 194.2 μM, respectively (Figure 5). These results suggest that AH-034/11365875 and AH-262/08373040 are moderate NA inhibitors.

The novelty of these two new NA inhibitors was also analyzed by calculating their structural similarity to the 281 previously reported inhibitors. The pairwise similarity of these inhibitors was calculated with the Tanimoto coefficient based on atom pair descriptors [38, 39]. The similarity of compounds AH-034/11365875 and AH-262/08373040 to the most similar compounds among the previously reported inhibitors was 0.281 and 0.373, respectively. Thus, these two compounds have very low similarity to previously reported inhibitors, suggesting that AH-034/11365875 and AH-262/08373040 are NA inhibitors with novel scaffolds. Because these two inhibitors were screened from a lead-like library, they can be considered lead compounds for the development of new anti-influenza drugs. Our results also confirm that our virtual screening strategy is capable of discovering new NA inhibitor scaffolds.

## DISCUSSION

In this study, we developed a machine-learning-based influenza-virus-NA-specific scoring function, called RF-NA-Score, using 67 NA–ligand complexes and their experimental binding affinities obtained from the PDBbind database as the training data. The performance of RF-NA-Score in predicting the binding affinities of ligands and NA was validated with 5-CV and LOOCV, and RF-NA-Score was more accurate than RF-Score. We also validated the accuracy of AutoDock, AutoDock Vina, and LeDock in docking ligands to influenza NA. We then evaluated various docking–rescoring virtual screening strategies using different docking software tools combined with different scoring methods, using a ligand dataset containing 281 NA inhibitors and 322 noninhibitors. The results suggest that rescoring with RF-NA-Score significantly improves the efficiency of virtual screening and that the best strategy involves docking with AutoDock, AutoDock Vina, or LeDock, rescoring with RF-NA-Score, and then averaging the scores. This strategy was then used to screen 52,631 lead-like compounds in the SPECS database. One hundred compounds were selected for testing in an *in vitro* H7N9 NA inhibition assay, and two compounds showed moderate anti-H7N9 NA activity. These two compounds were shown to be novel scaffolds, supporting the notable advantage of RF-NA-Score and this virtual screening strategy.

Despite the advantages of the virtual screening strategy proposed in this study, there are some noteworthy limitations. First, although we have validated the accuracy of AutoDock, AutoDock Vina, and LeDock in docking ligands to influenza NA, the docking software tools may still get incorrect results in docking other ligands, thus affecting the effectiveness of RF-NA-Score and the virtual screening strategy. Then, it needs to collect a large number of crystal structures of protein–ligand complexes as well as their experimental binding affinity values prior to the training of the machine-learning-based scoring function. But for most drug targets there is not enough data to establish a specific scoring function, which limits the scope of application of this strategy. Moreover, the proposed virtual screening strategy uses three docking software tools and a rescoring function to calculate binding affinity of a ligand, which requires significantly more computational power. If we only use the proposed strategy to evaluate the compounds that were top-ranked in traditional virtual screening, the computational cost should be significantly reduced, which should be further studied.

## MATERIALS AND METHODS

### Data preparation

The training dataset used to develop an influenza-virus-NA-specific scoring function for the prediction of ligand binding affinities was derived from the PDBbind database version 2016 [40], a comprehensive collection of protein–ligand complexes with high-quality crystal structures and experimentally measured binding affinity data. The binding affinity data are presented as −log (Kd/Ki/IC_50_) in the PDBbind database, which is a suitable format for training machine-learning-based scoring functions. In the present study, we searched the PDBbind database and collected 67 NA–ligand complexes with three-dimensional (3D) structures and binding affinity data. These 67 complexes were used as the training set for constructing the scoring function. The complete list of PDB codes and the corresponding binding affinities is provided in (Supplementary Table 1. The binding affinities of the 67 selected complexes ranged widely from 2.0 to 9.74, which implies that the ligands in the training dataset had diverse chemical structures.

To evaluate the performance of the scoring functions in the virtual screening process, comprehensive searches of the literature and the bindingDB database [41] were conducted to collect small molecules with known inhibitory activities (IC_50_ values) against influenza A virus NA. Because the aim of this study was to screen inhibitors of NA of the H7N9 influenza virus, and N9 belongs to the group 2 NAs (N2, N3, N6, N7, and N9) [42], we only collected the structures and corresponding IC_50_ values of group 2 NA inhibitors. In total, 706 molecules were finally collected, with IC_50_ values ranging from 0.1 nM to > 7 mM. Because the molecules were collected from multiple studies reported in the literature, the methods, test conditions, and sources of NA used for the NA inhibition assays were not exactly identical [43]. Therefore, there may have been some discrepancies in the IC_50_ value of these molecules. Consequently, we categorized the molecules with IC_50_ < 10 μM as inhibitors, and those with IC_50_ > 50 μM as noninhibitors. The molecules in the gray area (10 μM < IC_50_ < 50 μM) were removed to reduce the possible influence of variable IC_50_ values on the accuracy of our evaluation of the performance of the scoring functions [44-46]. Ultimately, the ligand dataset contained 281 inhibitors and 322 noninhibitors. The details of the ligand dataset are given in (Supplementary Table 2.

Because there were no experimentally determined 3D structures for these collected molecules when complexed with NA, we predicted them with the molecular docking method. This is also the method used when virtual screening is performed. The accuracy of the molecular docking software tools in predicting the NA–inhibitor complexes was confirmed by testing the ability of the programs to redock the ligand into the crystal structure of NA. Four inhibitors, oseltamivir carboxylate, zanamivir, laninamivir, and peramivir, were docked to the crystal structure of H7N9 NA (PDB ID: 4MWJ), and the resulting inhibitor–NA complexes were then compared with their experimentally determined complex structures (oseltamivir carboxylate, 4MWQ; zanamivir, 4MWR; laninamivir, 4MWU; and peramivir, 4MWV).

### Building an NA-specific scoring function

RF-Score is a machine-learning-based scoring function built with the random forests algorithm of Ballester and Mitchell [33, 34]. The recent version of RF-Score has outperformed 22 state-of-the-art scoring functions on the PDBbind benchmark [35]. In the present study, we trained a new implementation of RF-Score on a refined set of the PDBbind database 2016 (containing 3766 protein–ligand complexes), based on 36 RF-Score features and 11 Vina features. The NA-specific scoring function (RF-NA-Score) was trained on 67 NA–ligand complexes taken from the PDBbind database 2016 using the same method as was used to train RF-Score. The performance of RF-Score in predicting the binding affinity for NA was tested on the 67 NA–ligand complexes, and the performance of RF-NA-Score was rigorously validated with 5-fold cross-validation (5-CV), leave-one-out cross-validation (LOOCV), and resampling test, which have been commonly used in the the evaluation of machine learning models [29, 47-49]. In 5-CV, the 67 complexes were randomly divided into five equal subsets: four subsets were used as training sets to predict the binding affinities of the complexes in the remaining one subset. This process was repeated five times until each subset has been used as the test set. In LOOCV, for each run, just one sample was used as the test set, and the other samples were used as the training set, and this process was repeated until all the samples has been used as the test sample. In resampling test, 70% of the complexes were randomly sampled to training data to build the scoring function, and the remaining 30% were used to test the scoring function [50]. The test was repeated for 10 times.

The performance of the scoring functions was evaluated with RMSE, Pearson’s correlation coefficient (Rp), and Spearman’s rank correlation coefficient (Rs) between the predicted and measured binding affinities, because these are widely used to evaluate scoring functions [35, 51, 52]. RMSE represents the differences between the predicted and measured binding affinity values. Rp measures the linear correlation between the predicted and measured binding affinities, and Rs measures the rank correlation between the predicted and measured binding affinities. Rs shows how well the scoring function ranks the ligands according to their binding affinities.

### Molecular docking calculations

For molecular docking, the 3D structures of all the ligands used in this study were download from ZINC15 [53] or generated with CORINA Classic (https://www.mn-am.com/online_demos/corina_demo). AutoDock (version 4.2.6) [54], AutoDock Vina (version 1.1.2) [55], and LeDock (version 1.0) (http://www.lephar.com/) were used for molecular docking. The crystal structure of H7N9 NA (A/Anhui/1/2013) was download from the Protein Data Bank (PDB ID: 4MWJ) and used as the receptor in the docking process. The docking site of NA was determined based on the position of the co-crystalized ligand in the structure of the NA–oseltamivir carboxylate complex (PDB ID: 4MWQ). A description of each software and how it was used in this study is given briefly below.

### Autodock

AutoDock is a popular noncommercial protein–ligand docking program. The scoring function of AutoDock is a semi-empirical free-energy force field. To use AutoDock, the structure files of the receptor and ligands were processed with AutoDockTools in MGLTools (version 1.5.6) [54] to merge the nonpolar hydrogen, add Gasteiger atomic charges, and set atom type to the AutoDock-supported type. During docking, the Lamarckian genetic algorithm was used to optimize the ligand conformation and 10 docking runs were performed for each ligand. The population size was set to 150, the number of generations was set to 27,000, and the maximum number of energy evaluations was set to 2,500,000.

### Autodock vina

AutoDock Vina is a new docking software, offering multicore capability, high performance, and enhanced accuracy [55]. AutoDock Vina uses a new knowledge-based empirical scoring function and the same receptor and ligand files as AutoDock. We used the default values for all the parameters, except that the number of CPUs was set to 1.

### Ledock

LeDock is a newly released docking program based on a combination of simulated annealing and a genetic algorithm, using a physics/knowledge-based scoring scheme. LeDock displayed high accuracy and good speed in a recent comprehensive docking program evaluation study [56]. The protein is processed by LePro (http://www.lephar.com/) and the ligands do not require processing. All the parameters were set to the default values.

### Virtual screening strategy using molecular docking software and RF-NA-Score

**Figure 6:**
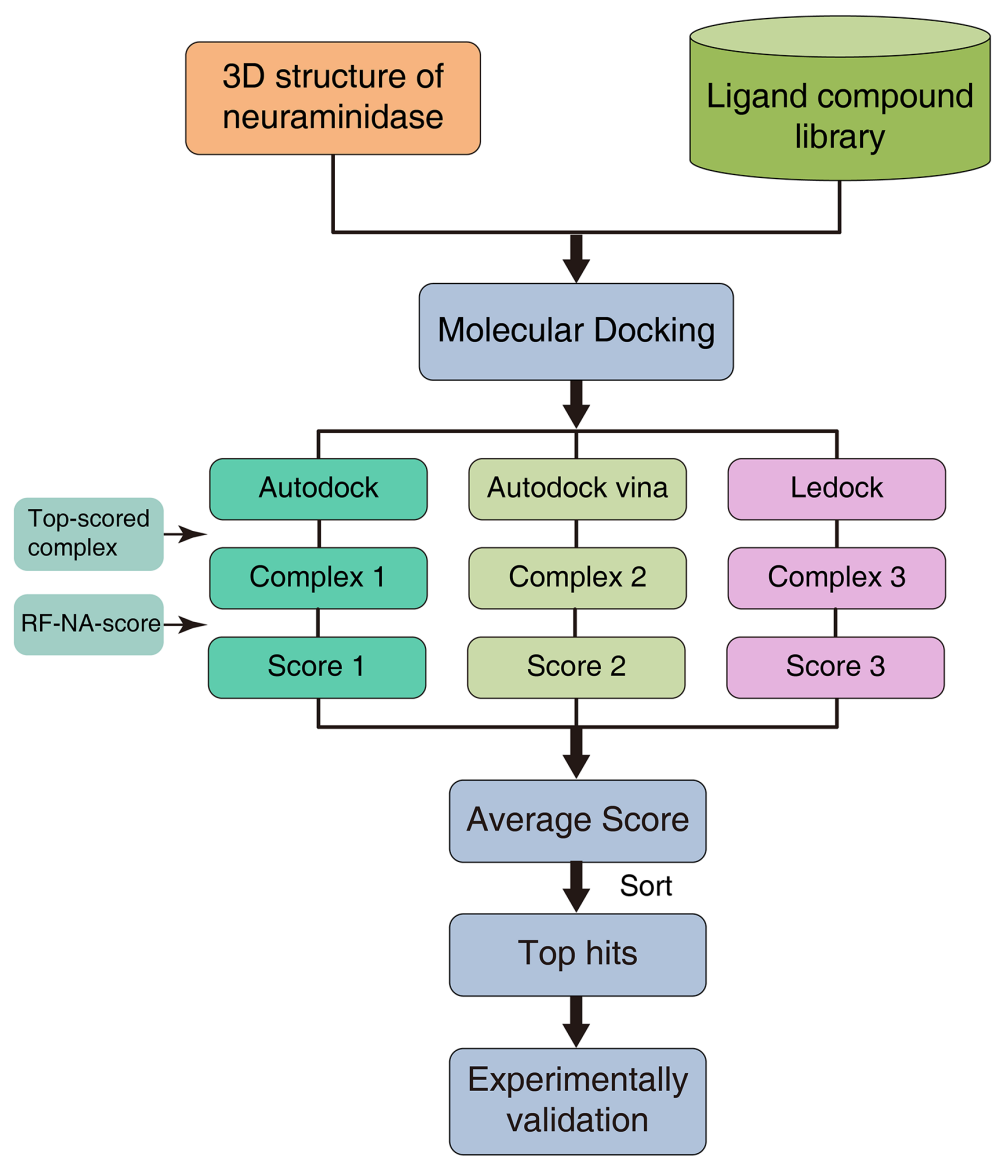
Virtual screening strategy using molecular docking software and RF-NA-Score

RF-NA-Score was used for virtual screening as illustrated in Figure 6. A molecular docking software, such as AutoDock, AutoDock Vina, or LeDock, was used to predict the complex structures of NA and the ligands in the compound library. The top-scoring binding conformations for each docking software were then selected as putative complex structures. A new score for each complex structure was then calculated with a rescoring function, such as RF-Score or RF-NA-Score. An average score was also computed by averaging the scores for the complex structures predicted by the different docking software tools. The ligands were then sorted according to their scores, and the ligands with higher scores were more likely to be NA inhibitors. This process can be called a “docking–rescoring strategy”.

The effectiveness of various virtual screening strategies using this docking–rescoring method, with different docking software tools combined with different rescoring functions, was evaluated based on the ligand dataset collected in this study.

The performance of the virtual screening strategies was evaluated with several methods, including score distributions, p values from student’s *t* test, enrichment factors, ROC curves and the areas under the ROC curves (AUCs). The score distributions of inhibitors and noninhibitors were presented as histograms and density curves. The student’s *t* test was performed to evaluate the statistical significance of the mean scores of the inhibitors and noninhibitors. Enrichment factor reflects the ability of the virtual screening method to enrich inhibitors in top-scored results. In this study, the enrichment factor at 10% (EF10) was calculated using the following formula [57]:

EF10=anAN (1)

where *a* is the number of actives (inhibitors) in top-scored 10% compounds, *n* is the number of compounds in 10% of the dataset (in this study *n*=60), *A* is the number of actives (inhibitors) in the dataset (here *A* = 281), *N* is the number of compounds in the dataset (here *N* = 603). The EF10 has an upper bound of 2.15. The ROC curves and the areas under the ROC curves (AUCs) can give more detail diagnose on performance of virtual screening method.

### Virtual screening of the SPECS database

The virtual screening strategy with the best performance was used to screen for influenza virus NA inhibitors in the SPECS database (http://www.specs.net). The 3D structures of 52,631 lead-like compounds (250 < molecular weight < 350, and logP < 3.5) in the SPECS database were downloaded from ZINC15. These compounds were subjected to the virtual screening workflow, and the 1000 top-ranking compounds were clustered by their structural similarities using the binning clustering algorithm in ChemMine tools [58], with a similarity cutoff of 0.4. To increase the diversity of the candidate structures, only one compound in each cluster, with the best score, was selected for bioassay validation. Because some clusters contained many compounds, we selected slightly more compounds from these clusters. Finally, 100 candidate compounds were selected and purchased from SPECS.

### *In vitro* NA inhibition assay

The *in vitro* inhibitory activities of the candidate compounds against NA were assayed with the modified method of Potier et al. [59], using oseltamivir carboxylate (MedChem Express, HY-13318) as the positive control. 2′-(4-Methylumbelliferyl)-α-D-N-acetylneuraminic acid (4MU-NANA, Sigma, M8639) in MES (Sigma, M8250) buffer (32.5 mM MES, 4 mM CaCl_2_, pH 6.5) was used as the substrate and NA from A/Anhui/1/2013(H7N9) (Sino Biological Inc., 40108-VNAHC) in MES buffer was used as the enzyme. The candidate compounds were dissolved in DMSO and diluted to 500 μM in MES buffer. For the assay, 10 μL of the compound sample solution was mixed with 10 μL of NA in a 96-well microplate and incubated for 30 min at 37 °C. Then 30 μL of 100 μM MUNANA was added and incubated for 60 min at 37 °C, after which 150 μL of stop solution (0.1 M glycine, 25% ethanol, pH 10.7) was added to each well to terminate the reaction. The fluorogenic end-product 4-methylumbelliferone (4-MU) was detected with a SpectraMax M5 microplate spectrophotometer (Molecular Devices, Sunnyvale, CA, United States) at an excitation wavelength of 355 nm and an emission wavelength of 460 nm. Relative fluorescence units (RFU) were calculated by subtracting the background values. The inhibition rate (IR) was calculated with the formula: IR (%) = (RFU_DMSO_ − RFU_sample_)/RFU_DMSO_ × 100. IC_50_ was calculated by plotting IR against the compound concentration, using GraphPad PRISM 7. The assays for each compound were performed in triplicate.

## SUPPLEMENTARY MATERIALS TABLES






