# Supplementary material for: Virtual screening approach to identifying influenza virus neuraminidase inhibitors using molecular docking combined with machine-learning-based scoring function
Source: Oncotarget. 2017 Sep 15;8(47):83142–54. doi: 10.18632/oncotarget.20915 (PMC5669956; doi:10.18632/oncotarget.20915)
Supplement: Supplementary file 1 [file oncotarget-08-83142-s001.pdf]

Virtual screening approach to identifying influenza virus neuraminidase inhibitors using molecular docking combined with machine-learning-based scoring function

SUPPLEMENTARY MATERIALS

Supplementary Table 1: List of 67 neuraminidase-ligand complex used as the training set for scoring function building

See Supplementary File 1

Supplementary Table 2: Details of the 281 inhibitors and 322 non-inhibitors collected from literatures and bindingDB database

See Supplementary File 2
