# Supplementary material for: Virtual screening approach to identifying influenza virus neuraminidase inhibitors using molecular docking combined with machine-learning-based scoring function
Source: Oncotarget. 2017 Sep 15;8(47):83142–54. doi: 10.18632/oncotarget.20915 (PMC5669956; doi:10.18632/oncotarget.20915)
Supplement: Supplementary file 2 [file oncotarget-08-83142-s002.docx]

**Supplementary Table 1:** **List of 67 neuraminidase-ligand complex used as the training set for scoring function building**

| **Index** | **PDB ID** | **Binding affinity**  **-log(Kd/Ki/IC50)** | **Kd/Ki/IC50** | **Resolution (Å)** | **Release year** |
| --- | --- | --- | --- | --- | --- |
| 1 | 1ing | 2.00 | IC50>10mM | 2.4 | 1996 |
| 2 | 1inh | 2.30 | IC50=5mM | 2.4 | 1996 |
| 3 | 2qwb | 2.74 | Ki=1820uM | 2.0 | 1998 |
| 4 | 1b9s | 3.18 | IC50>667uM | 2.5 | 1999 |
| 5 | 4gzx | 3.19 | Kd=645uM | 2.5 | 2012 |
| 6 | 1f8d | 3.40 | Ki=400uM | 1.4 | 2001 |
| 7 | 2qwc | 3.55 | Ki=280uM | 1.6 | 1998 |
| 8 | 2qwg | 3.64 | IC50=230uM | 1.8 | 1998 |
| 9 | 1b9v | 3.65 | IC50=224uM | 2.4 | 1999 |
| 10 | 4mww | 3.67 | IC50=214770nM | 1.9 | 2013 |
| 11 | 4gzw | 4.52 | Kd=30.0uM | 2.5 | 2012 |
| 12 | 1vcj | 4.59 | IC50=26uM | 2.4 | 2004 |
| 13 | 1f8e | 4.82 | Ki=15uM | 1.4 | 2001 |
| 14 | 2qwd | 4.85 | Ki=14uM | 2.0 | 1998 |
| 15 | 1nnb | 5.00 | Ki=10uM | 2.8 | 1994 |
| 16 | 1inf | 5.00 | IC50=10uM | 2.4 | 1996 |
| 17 | 4dgr | 5.04 | IC50=9.1uM | 1.6 | 2012 |
| 18 | 1b9t | 5.10 | IC50=8uM | 2.4 | 1999 |
| 19 | 1f8b | 5.40 | Ki=4.0uM | 1.8 | 2001 |
| 20 | 1a4q | 5.44 | IC50=3.6uM | 1.9 | 1999 |
| 21 | 2qwf | 5.67 | Ki=2.160uM | 1.9 | 1998 |
| 22 | 3ti4 | 6.02 | IC50=947nM | 1.6 | 2011 |
| 23 | 4cpy | 6.31 | Ki=491.35nM | 1.8 | 2014 |
| 24 | 4ks3 | 6.37 | Ki=430nM | 2.6 | 2013 |
| 25 | 1xog | 6.39 | IC50=410nM | 2.8 | 2005 |
| 26 | 4d8s | 6.49 | IC50=0.32uM | 2.4 | 2013 |
| 27 | 4mx0 | 6.73 | IC50=184.7nM | 2.1 | 2013 |
| 28 | 3tib | 6.89 | IC50=129nM | 2.2 | 2011 |
| 29 | 4ks5 | 6.89 | Ki=130nM | 2.7 | 2013 |
| 30 | 4mwy | 7.05 | IC50=89.6nM | 1.8 | 2013 |
| 31 | 3cl0 | 7.07 | Ki=84.8nM | 2.2 | 2008 |
| 32 | 3k3a | 7.08 | Ki=84.0nM | 2.6 | 2010 |
| 33 | 4mwx | 7.12 | IC50=75.7nM | 1.8 | 2013 |
| 34 | 4ks4 | 7.14 | Ki=72nM | 2.5 | 2013 |
| 35 | 1xoe | 7.39 | IC50=41nM | 2.2 | 2005 |
| 36 | 1f8c | 7.40 | Ki=0.04uM | 1.7 | 2001 |
| 37 | 2qwe | 7.48 | Ki=0.033uM | 2.0 | 1998 |
| 38 | 3cl2 | 7.59 | Ki=25.9nM | 2.5 | 2008 |
| 39 | 4cpz | 7.68 | Ki=20.85nM | 2.2 | 2014 |
| 40 | 4gzt | 7.77 | Ki=17.0nM | 2.2 | 2012 |
| 41 | 4gzp | 7.84 | Ki=14.3nM | 2.3 | 2012 |
| 42 | 4b7j | 7.95 | Ki=11.1nM | 2.4 | 2012 |
| 43 | 4hzz | 8.08 | IC50=8.31nM | 1.6 | 2013 |
| 44 | 3k39 | 8.08 | Ki=8.3nM | 2.5 | 2010 |
| 45 | 1a4g | 8.40 | IC50=0.004uM | 2.2 | 1999 |
| 46 | 4mwu | 8.49 | IC50=3.24nM | 1.8 | 2013 |
| 47 | 3tia | 8.51 | IC50=3.12nM | 1.8 | 2011 |
| 48 | 1bji | 8.70 | IC50=2nM | 2.0 | 1998 |
| 49 | 3ckz | 8.72 | Ki=1.9nM | 1.9 | 2008 |
| 50 | 3ti3 | 8.74 | IC50=1.83nM | 1.8 | 2011 |
| 51 | 4b7n | 8.78 | Ki=1.65nM | 2.8 | 2012 |
| 52 | 4ks1 | 8.82 | Ki=1.5nM | 2.2 | 2013 |
| 53 | 3tic | 8.87 | IC50=1.36nM | 1.9 | 2011 |
| 54 | 4hzw | 8.88 | IC50=1.31nM | 1.7 | 2013 |
| 55 | 3k37 | 8.92 | Ki=1.2nM | 2.0 | 2010 |
| 56 | 3ti5 | 8.95 | IC50=1.11nM | 1.9 | 2011 |
| 57 | 4m3m | 9.05 | IC50=0.9nM | 2.1 | 2014 |
| 58 | 3ti8 | 9.05 | IC50=0.90nM | 1.6 | 2011 |
| 59 | 4qn7 | 9.08 | IC50=0.84nM | 2.3 | 2014 |
| 60 | 4mwq | 9.10 | IC50=0.79nM | 2.0 | 2013 |
| 61 | 3ti6 | 9.27 | IC50=0.54nM | 1.7 | 2011 |
| 62 | 4hzx | 9.30 | IC50=0.5nM | 2.2 | 2013 |
| 63 | 4ks2 | 9.34 | Ki=0.46nM | 2.6 | 2013 |
| 64 | 4mwr | 9.39 | IC50=0.41nM | 1.8 | 2013 |
| 65 | 4mwv | 9.40 | IC50=0.40nM | 2.0 | 2013 |
| 66 | 4b7r | 9.64 | Ki=0.23nM | 1.9 | 2012 |
| 67 | 4b7q | 9.74 | Ki=0.18nM | 2.7 | 2012 |
