# Supplementary material for: Virtual screening approach to identifying influenza virus neuraminidase inhibitors using molecular docking combined with machine-learning-based scoring function
Source: Oncotarget. 2017 Sep 15;8(47):83142–54. doi: 10.18632/oncotarget.20915 (PMC5669956; doi:10.18632/oncotarget.20915)
Supplement: Supplementary file 3 [file oncotarget-08-83142-s003.docx]

**Supplementary Table 2:** Details of the 281 inhibitors and 322 non-inhibitors collected from literatures and bindingDB database

| **Index** | **IC50** | **Category** | **SMILES** | **Reference** |
| --- | --- | --- | --- | --- |
| 1 | 330 | Inhibitor | CCCCC1=NN=C(N1CCC)[C@H]2[C@@H]([C@H](C=C(O2)C(=O)O)N)NC(=O)C | doi:10.1016/S0960-894X(97)00399-5 |
| 2 | 56000 | Non-inhibitor | CCCCC1=NOC(=N1)[C@H]2[C@@H]([C@H](C=C(O2)C(=O)O)N)NC(=O)C | doi:10.1016/S0960-894X(97)00399-5 |
| 3 | 2 | Inhibitor | CC(=O)N[C@@H]1[C@H](C=C(O[C@H]1[C@@H]([C@@H](CO)O)O)C(=O)O)N=C(N)N | doi:10.1016/S0960-894X(97)00399-5 |
| 4 | 2 | Inhibitor | CCCN(CCC1=CC=CC=C1)C(=O)[C@H]2[C@@H]([C@H](C=C(O2)C(=O)O)N)NC(=O)C | doi:10.1016/S0960-894X(97)00399-5 |
| 5 | 99000 | Non-inhibitor | CC(=O)N[C@@H]1[C@H](C=C(O[C@H]1C2=NC(=NO2)CC3=CC=CC=C3)C(=O)O)N | doi:10.1016/S0960-894X(97)00399-5 |
| 6 | 87000 | Non-inhibitor | CC(=O)N[C@@H]1[C@H](C=C(O[C@H]1C2=NNC(=N2)CC3=CC=CC=C3)C(=O)OC(C4=CC=CC=C4)C5=CC=CC=C5)NC(=O)OC(C)(C)C | doi:10.1016/S0960-894X(97)00399-5 |
| 7 | 190000 | Non-inhibitor | CC(=O)N[C@@H]1[C@H](C=C(O[C@H]1C(=O)NC)C(=O)O)N | doi:10.1016/S0960-894X(96)00542-2 |
| 8 | 5 | Inhibitor | CCCN(CCC1=CC=CC=C1)C(=O)[C@H]2[C@@H]([C@H](C=C(O2)C(=O)O)N=C(N)N)NC(=O)C | doi:10.1016/S0960-894X(96)00542-2 |
| 9 | 7000 | Inhibitor | CC(=O)N[C@@H]1[C@H](C=C(O[C@H]1C(=O)NC)C(=O)O)N=C(N)N | doi:10.1016/S0960-894X(96)00542-2 |
| 10 | 16 | Inhibitor | CCCCN(CCCC)C(=O)[C@H]1[C@@H]([C@H](C=C(O1)C(=O)O)N=C(N)N)NC(=O)C | doi:10.1016/S0960-894X(96)00542-2 |
| 11 | 300 | Inhibitor | CC(=O)N[C@@H]1[C@H](C=C(O[C@H]1[C@@H]([C@@H](CO)O)O)C(=O)O)N | doi:10.1016/S0960-894X(96)00542-2 |
| 12 | 3 | Inhibitor | CCCN(CCC1=CC=C(C=C1)C2=CC=CC=C2)C(=O)[C@H]3[C@@H]([C@H](C=C(O3)C(=O)O)N)NC(=O)C | doi:10.1016/S0960-894X(96)00542-2 |
| 13 | 2400 | Inhibitor | CC(=O)N[C@@H]1[C@H](C=C(O[C@H]1C(=O)N(C)C)C(=O)O)N | doi:10.1016/S0960-894X(96)00542-2 |
| 14 | 180 | Inhibitor | CCCN(C)C(=O)[C@H]1[C@@H]([C@H](C=C(O1)C(=O)O)N)NC(=O)C | doi:10.1016/S0960-894X(96)00542-2 |
| 15 | 3 | Inhibitor | CCCN(CCC)C(=O)[C@H]1[C@@H]([C@H](C=C(O1)C(=O)O)N)NC(=O)C | doi:10.1016/S0960-894X(96)00542-2 |
| 16 | 450 | Inhibitor | CCCCN(CCCC)C(=O)[C@H]1[C@@H]([C@H](C=C(O1)C(=O)O)N)NC(=O)C | doi:10.1016/S0960-894X(96)00542-2 |
| 17 | 3 | Inhibitor | CCN(CC)C(=O)[C@H]1[C@@H]([C@H](C=C(O1)C(=O)O)N)NC(=O)C | doi:10.1016/S0960-894X(96)00542-2 |
| 18 | 3 | Inhibitor | CCCN(CC)C(=O)[C@H]1[C@@H]([C@H](C=C(O1)C(=O)O)N)NC(=O)C | doi:10.1016/S0960-894X(96)00542-2 |
| 19 | 4 | Inhibitor | CCCCN(CCC)C(=O)[C@H]1[C@@H]([C@H](C=C(O1)C(=O)O)N)NC(=O)C | doi:10.1016/S0960-894X(96)00542-2 |
| 20 | 23 | Inhibitor | CCCCCCCCCN(CCC)C(=O)[C@H]1[C@@H]([C@H](C=C(O1)C(=O)O)N)NC(=O)C | doi:10.1016/S0960-894X(96)00542-2 |
| 21 | 320 | Inhibitor | CC(=O)N[C@@H]1[C@H](C=C(O[C@H]1C(=O)N(C)CCC2=CC=CC=C2)C(=O)O)N | doi:10.1016/S0960-894X(96)00542-2 |
| 22 | 5 | Inhibitor | CCN(CCC1=CC=CC=C1)C(=O)[C@H]2[C@@H]([C@H](C=C(O2)C(=O)O)N)NC(=O)C | doi:10.1016/S0960-894X(96)00542-2 |
| 23 | 500 | Inhibitor | CCCNC(=O)[C@H]1[C@@H]([C@H](C=C(O1)C(=O)O)N=C(N)N)NC(=O)C | doi:10.1016/S0960-894X(96)00542-2 |
| 24 | 25 | Inhibitor | CC(=O)N[C@@H]1[C@H](C=C(O[C@H]1C(=O)N(C)C)C(=O)O)N=C(N)N | doi:10.1016/S0960-894X(96)00542-2 |
| 25 | 1.4 | Inhibitor | CCC(CC)[C@@H]([C@H]1[C@@H](C[C@@H]([C@H]1O)C(=O)O)N=C(N)N)NC(=O)C | doi:10.1021/jm0002679 |
| 26 | 130000 | Non-inhibitor | CCN(C(C)C)C(=O)N1C[C@H]([C@H]([C@@H]1CNS(=O)(=O)C)N)C(=O)O | doi:10.1021/jm000468c |
| 27 | 96000 | Non-inhibitor | CCN(C(C)C)C(=O)N1C[C@H]([C@H]([C@@H]1CNC(=O)C=C)N)C(=O)O | doi:10.1021/jm000468c |
| 28 | 280 | Inhibitor | CCN(C(C)C)C(=O)N1C[C@H]([C@H]([C@@H]1CNC(=O)C(F)(F)F)N)C(=O)O | doi:10.1021/jm000468c |
| 29 | 7500 | Inhibitor | CCN(C(C)C)C(=O)N1C[C@H]([C@H]([C@@H]1CNC(=O)C)N)C(=O)O | doi:10.1021/jm000468c |
| 30 | 4000 | Inhibitor | CC(C)N(C(C)C)C(=O)N1C[C@H]([C@H](C1)N)C(=O)O | doi:10.1021/jm000468c |
| 31 | 1300 | Inhibitor | CC(C)N(CCCCC(=O)O)C(=O)N1C[C@H]([C@H](C1)N)C(=O)O | doi:10.1021/jm000468c |
| 32 | 50000 | Non-inhibitor | CC(C)(C)OC(=O)N1C[C@@H]([C@@H](C1)N)C(=O)O | doi:10.1021/jm000468c |
| 33 | 1300 | Inhibitor | CC(C)N(CCC1=CC=CC=N1)C(=O)N2C[C@H]([C@H](C2)N)C(=O)O | doi:10.1021/jm000468c |
| 34 | 2000 | Inhibitor | CC(C)N(CCCCCO)C(=O)N1C[C@H]([C@H](C1)N)C(=O)O | doi:10.1021/jm000468c |
| 35 | 1600 | Inhibitor | CCN(C(C)C)C(=O)N1C[C@H]([C@H](C1)N)C(=O)O | doi:10.1021/jm000468c |
| 36 | 2100 | Inhibitor | CC(C)N(CCCO)C(=O)N1C[C@H]([C@H](C1)N)C(=O)O | doi:10.1021/jm000468c |
| 37 | >50000 | Non-inhibitor | CN(C(N1C[C@H]([C@H](C1)N)C(=O)O)=O)CC | doi:10.1021/jm000468c |
| 38 | >50000 | Non-inhibitor | C(CN(CC(C)C)C(=O)N1C[C@H]([C@H](C1)N)C(=O)O)(C)C | doi:10.1021/jm000468c |
| 39 | >50000 | Non-inhibitor | CC(C)N(C(C)C)C(=O)N1C[C@@H]([C@@H](C1)NC(=O)N(C)C)C(=O)O | doi:10.1021/jm000468c |
| 40 | >50000 | Non-inhibitor | CCCCN[C@@H]1CN(C[C@@H]1C(=O)O)C(=O)N(C(C)C)C(C)C | doi:10.1021/jm000468c |
| 41 | >50000 | Non-inhibitor | CC(C)N(C(C)C)C(=O)N1C[C@@H]([C@@H](C1)NC)C(=O)O | doi:10.1021/jm000468c |
| 42 | >50000 | Non-inhibitor | CC(C)N(C(C)C)C(=O)N1C[C@@H]([C@@H](C1)NCC2=CC=CC=C2)C(=O)O | doi:10.1021/jm000468c |
| 43 | >50000 | Non-inhibitor | CC(C)N(C(C)C)C(=O)N1C[C@@H]([C@@H](C1)NC(=O)C(C)N)C(=O)O | doi:10.1021/jm000468c |
| 44 | >50000 | Non-inhibitor | CCC(=O)NC[C@H]1[C@@H]([C@@H](CN1C(=O)N(CC)C(C)C)C(=O)O)N | doi:10.1021/jm000468c |
| 45 | 410 | Inhibitor | CC(C)C[C@@H]([C@H]1[C@@H](C[C@@H](O1)C(=O)O)C(=O)OC)NC(=O)C | doi:10.1016/j.bmcl.2004.10.022 |
| 46 | 580 | Inhibitor | CC(C)C[C@@H]([C@H]1[C@@H](C[C@@H](O1)C(=O)O)C2=CN=CN2)NC(=O)C | doi:10.1016/j.bmcl.2004.10.022 |
| 47 | 2.9 | Inhibitor | CCC(CC)O[C@@H]1C=C(C[C@@H]([C@H]1NC(=O)C)N)C(=O)O | doi:10.1021/jm3008486 |
| 48 | 14 | Inhibitor | CC(C)CN(CCC1=CC=CC=C1)C(=O)[C@H]2[C@@H]([C@H](C=C(O2)C(=O)O)N)NC(=O)C | doi:10.1021/jm9703754 |
| 49 | 1000000 | Non-inhibitor | CC(=O)N[C@@H]1[C@H](C[C@](O[C@H]1[C@@H]([C@@H](CO)O)O)(C(=O)O)O)O | doi:10.1021/jm9703754 |
| 50 | 2600000 | Non-inhibitor | C1CC(=O)N(C1CN)C2=C(C=C(C=C2)C(=O)O)N=C(N)N | doi:10.1021/jm980707k |
| 51 | 48 | Inhibitor | CCC(CC)NC1=C(C=CC(=C1)C(=O)O)N2C(=O)CCC2(CO)CO | doi:10.1021/jm980707k |
| 52 | 760000 | Non-inhibitor | C1CC(N(C1=O)C2=CC=C(C=C2)C(=O)O)(CO)CO | doi:10.1021/jm980707k |
| 53 | 5000 | Inhibitor | C1CC(N(C1=O)C2=C(C=C(C=C2)C(=O)O)N=C(N)N)(CO)CO | doi:10.1021/jm980707k |
| 54 | 222000 | Non-inhibitor | CCC(CC)NC1=C(C=CC(=C1)C(=O)O)N2CCCC2=O | doi:10.1021/jm980707k |
| 55 | 10000 | Inhibitor | CC(=O)N[C@H]1[C@@H](C=C(O[C@@H]1[C@@H]([C@@H](CO)O)O)C(=O)O)O | doi:10.1021/jm980707k |
| 56 | 10000 | Inhibitor | CC(=O)N[C@H]1[C@@H](C[C@](O[C@@H]1[C@@H]([C@@H](CO)O)O)(C(=O)O)O)O | doi:10.1021/jm980707k |
| 57 | 200 | Inhibitor | CCC(CC)CC1=C(C=CC(=C1)C(=O)O)N2C(=O)CCC2(CO)CO | doi:10.1016/j.bmc.2012.05.001 |
| 58 | 200 | Inhibitor | CCC(C)OCC1=C(C=CC(=C1)C(=O)O)N2C(=O)CCC2(CO)CO | doi:10.1016/j.bmc.2012.05.001 |
| 59 | 3000 | Inhibitor | CC(C)CC1=C(C=CC(=C1)C(=O)O)N2C(=O)CCC2(CO)CO | doi:10.1016/j.bmc.2012.05.001 |
| 60 | 500 | Inhibitor | CCC(CC)OCC1=C(C=CC(=C1)C(=O)O)N2C(=O)CCC2(CO)CO | doi:10.1016/j.bmc.2012.05.001 |
| 61 | 4000000 | Non-inhibitor | CC(C)OCC1=C(C=CC(=C1)C(=O)O)N2C(=O)CCC2(CO)CO | doi:10.1016/j.bmc.2012.05.001 |
| 62 | 410 | Inhibitor | CCC(=CC1=C(C=CC(=C1)C(=O)O)N2C(=O)CCC2(CO)CO)CC | doi:10.1016/j.bmc.2012.05.001 |
| 63 | 200000 | Non-inhibitor | C1(=CC=C(C(=C1)COCC2=CC=CC=C2)N3C(CCC3(CO)CO)=O)C(=O)O | doi:10.1016/j.bmc.2012.05.001 |
| 64 | 500 | Inhibitor | C1(=CC=C(C(=C1)CC(CCC)CCC)N2C(CCC2(CO)CO)=O)C(=O)O | doi:10.1016/j.bmc.2012.05.001 |
| 65 | 0.7 | Inhibitor | CCCN(CCC1=CC=CC=C1)C(=O)[C@H]2[C@@H]([C@H](C=C(O2)C(=O)O)N)NC(=O)CC | doi:10.1016/S0960-894X(01)00019-1 |
| 66 | 7 | Inhibitor | CCCN(CCC1=CC=CC=C1)C(=O)[C@H]2[C@@H]([C@H](C=C(O2)C(=O)O)O)NC(=O)C | doi:10.1016/S0960-894X(01)00019-1 |
| 67 | 420 | Inhibitor | CCCN(CCC)C(=O)[C@H]1[C@@H]([C@H](C=C(O1)C(=O)O)NCC)NC(=O)C | doi:10.1016/S0960-894X(01)00019-1 |
| 68 | 5 | Inhibitor | CCCN(CCC)C(=O)[C@H]1[C@@H]([C@H](C=C(O1)C(=O)O)N)NC(=O)CC | doi:10.1016/S0960-894X(01)00019-1 |
| 69 | 0.3 | Inhibitor | CCCN(CCC)C(=O)[C@H]1[C@@H]([C@H](C=C(O1)C(=O)O)N)NC(=O)C(F)(F)F | doi:10.1016/S0960-894X(01)00019-1 |
| 70 | 140 | Inhibitor | CCCN(CCC)C(=O)[C@H]1[C@@H]([C@@H](C=C(O1)C(=O)O)N)NC(=O)C | doi:10.1016/S0960-894X(01)00019-1 |
| 71 | 50 | Inhibitor | CCCN(CCC1=CC=CC=C1)C(=O)[C@H]2[C@@H]([C@H](C=C(O2)C(=O)O)N)NC(=O)C3CC3 | doi:10.1016/S0960-894X(01)00019-1 |
| 72 | 3000 | Inhibitor | CCCN(CCC)C(=O)[C@H]1[C@@H]([C@H](C=C(O1)C(=O)O)N)NS(=O)(=O)C | doi:10.1016/S0960-894X(01)00019-1 |
| 73 | 3 | Inhibitor | CCCN(CCC1=CC=CC=C1)C(=O)[C@H]2[C@@H]([C@H](C=C(O2)C(=O)O)N)NC(=O)C(F)(F)F | doi:10.1016/S0960-894X(01)00019-1 |
| 74 | 7 | Inhibitor | CCCN(CCC1=CC=CC=C1)C(=O)[C@H]2[C@@H](CC=C(O2)C(=O)O)NC(=O)C | doi:10.1016/S0960-894X(01)00019-1 |
| 75 | 17 | Inhibitor | CCCN(CCC1=CC=CC=C1)C(=O)[C@H]2[C@@H]([C@@H](C=C(O2)C(=O)O)O)NC(=O)C | doi:10.1016/S0960-894X(01)00019-1 |
| 76 | 240 | Inhibitor | CCCN(CCC)C(=O)[C@H]1[C@@H]([C@H](C=C(O1)C(=O)O)NC)NC(=O)C | doi:10.1016/S0960-894X(01)00019-1 |
| 77 | 1500000 | Non-inhibitor | C1=CC=C(C=C1)C(=O)NC2=CC=C(C=C2)C(=O)O | doi:10.1021/jm970479e |
| 78 | 2500000 | Non-inhibitor | C1=CC(=CC=C1C(=O)O)S(=O)(=O)N | doi:10.1021/jm970479e |
| 79 | 4000000 | Non-inhibitor | C1=CC(=CC(=C1)NC#N)C(=O)O | doi:10.1021/jm970479e |
| 80 | 5000000 | Non-inhibitor | C1=CC(=CC(=C1)N=C(N)N)C(=O)O | doi:10.1021/jm970479e |
| 81 | 2500 | Inhibitor | CC(=O)NC1=C(C=C(C=C1)C(=O)O)N=C(N)N | doi:10.1021/jm970479e |
| 82 | 2500000 | Non-inhibitor | CC(=O)NC1=C(C=C(C=C1)C(=O)O)CC[N+](=O)[O-] | doi:10.1021/jm970479e |
| 83 | 1000000 | Non-inhibitor | CC(C)C(=O)NC1=C(C=C(C=C1)C(=O)O)N=C(N)N | doi:10.1021/jm970479e |
| 84 | 5000 | Inhibitor | CNC(=O)C1=C(C=C(C=C1)C(=O)O)N=C(N)N | doi:10.1021/jm970479e |
| 85 | 9000 | Inhibitor | C1=CC(=C(C=C1C(=O)O)N=C(N)N)S(=O)(=O)N | doi:10.1021/jm970479e |
| 86 | 140000 | Non-inhibitor | CS(=O)CC1=C(C=C(C=C1)C(=O)O)N=C(N)N | doi:10.1021/jm970479e |
| 87 | 300000 | Non-inhibitor | CS(=O)(=O)CC1=C(C=C(C=C1)C(=O)O)N=C(N)N | doi:10.1021/jm970479e |
| 88 | 2000000 | Non-inhibitor | C1=C(C=C(C=C1N=C(N)N)N)C(=O)O | doi:10.1021/jm970479e |
| 89 | 1500000 | Non-inhibitor | C1=C(C=C(C=C1N=C(N)N)N=C(N)N)C(=O)O | doi:10.1021/jm970479e |
| 90 | 500000 | Non-inhibitor | C1=C(C=C(C=C1C(=O)O)N=C(N)N)/C=N\O | doi:10.1021/jm970479e |
| 91 | >7000000 | Non-inhibitor | C1=CC=C(C=C1)C(=O)O | doi:10.1021/jm970479e |
| 92 | >7000000 | Non-inhibitor | C1=CC(=CC=C1C(=O)O)N | doi:10.1021/jm970479e |
| 93 | >7000000 | Non-inhibitor | C1=CC(=CC=C1C(=O)O)O | doi:10.1021/jm970479e |
| 94 | >7000000 | Non-inhibitor | C1=CC(=CC=C1C(=O)O)N=C(N)N | doi:10.1021/jm970479e |
| 95 | >7000000 | Non-inhibitor | CC(=O)NC1=CC=C(C=C1)S(=O)(=O)N | doi:10.1021/jm970479e |
| 96 | >7000000 | Non-inhibitor | C1=CC(=CC(=C1)C(=O)O)C#N | doi:10.1021/jm970479e |
| 97 | >7000000 | Non-inhibitor | C1=CC(=CC(=C1)O)C(=O)O | doi:10.1021/jm970479e |
| 98 | >7000000 | Non-inhibitor | C1=CC(=CC(=C1)N)C(=O)O | doi:10.1021/jm970479e |
| 99 | >7000000 | Non-inhibitor | CNC(=O)NC1=CC=C(C=C1)C(=O)O | doi:10.1021/jm970479e |
| 100 | >7000000 | Non-inhibitor | C1=CC=C(C=C1)S(=O)(=O)NC2=CC=C(C=C2)C(=O)O | doi:10.1021/jm970479e |
| 101 | >7000000 | Non-inhibitor | C1=CC(=CC=C1C(=O)O)NC(=O)C(=O)O | doi:10.1021/jm970479e |
| 102 | >7000000 | Non-inhibitor | CC(=O)NC1=C(C=C(C=C1)C(=O)O)O | doi:10.1021/jm970479e |
| 103 | >7000000 | Non-inhibitor | CC(=O)NC1=CC=C(C=C1)C(=O)O | doi:10.1021/jm970479e |
| 104 | >7000000 | Non-inhibitor | CC(=O)NC1=CC=CC(=C1)C(=O)O | doi:10.1021/jm970479e |
| 105 | >7000000 | Non-inhibitor | CC(=O)NC1=CC=C(C=C1)S(=O)(=O)O | doi:10.1021/jm970479e |
| 106 | >7000000 | Non-inhibitor | C1=CC(=CC=C1C(CO)CO)C(=O)O | doi:10.1021/jm970479e |
| 107 | >7000000 | Non-inhibitor | C1=CC(=CC=C1C(=O)O)NC(=O)N | doi:10.1021/jm970479e |
| 108 | >7000000 | Non-inhibitor | CC(=O)NC1=CC=C(C=C1)[N+](=O)[O-] | doi:10.1021/jm970479e |
| 109 | >7000000 | Non-inhibitor | C1=CC(=CC=C1C(=O)N)C(=O)O | doi:10.1021/jm970479e |
| 110 | >7000000 | Non-inhibitor | CNC(=O)C1=CC=C(C=C1)C(=O)O | doi:10.1021/jm970479e |
| 111 | >7000000 | Non-inhibitor | CC(C1=CC=C(C=C1)C(=O)O)O | doi:10.1021/jm970479e |
| 112 | >7000000 | Non-inhibitor | C1=C(C=C(C=C1N)N)C(=O)O | doi:10.1021/jm970479e |
| 113 | >7000000 | Non-inhibitor | C1=CC(=CC(=C1)CCN=C(N)N)C(=O)O | doi:10.1021/jm970479e |
| 114 | >7000000 | Non-inhibitor | C/C(=N\N=C(N)N)/C1=CC=C(C=C1)C(=O)O | doi:10.1021/jm970479e |
| 115 | >7000000 | Non-inhibitor | COC(=O)NC1=CC=C(C=C1)C(=O)O | doi:10.1021/jm970479e |
| 116 | >7000000 | Non-inhibitor | C1=CC(=CC(=C1)NC(=S)N)C(=O)O | doi:10.1021/jm970479e |
| 117 | >7000000 | Non-inhibitor | CC(=O)NC1=C(C=C(C=C1)C(=O)O)CC(=O)O | doi:10.1021/jm970479e |
| 118 | >7000000 | Non-inhibitor | C1=CC(=CC=C1CCO)C(=O)O | doi:10.1021/jm970479e |
| 119 | >7000000 | Non-inhibitor | CC(=O)C1=CC=C(C=C1)C(=O)O | doi:10.1021/jm970479e |
| 120 | >7000000 | Non-inhibitor | C/C(=N\O)/C1=CC=C(C=C1)C(=O)O | doi:10.1021/jm970479e |
| 121 | >7000000 | Non-inhibitor | CC(=O)NC1=CC=C(C=C1)C2N=NN=N2 | doi:10.1021/jm970479e |
| 122 | >7000000 | Non-inhibitor | CNS(=O)(=O)C1=CC=C(C=C1)C(=O)O | doi:10.1021/jm970479e |
| 123 | >7000000 | Non-inhibitor | CC(=O)NC1=CC=C(C=C1)S(=O)[O-] | doi:10.1021/jm970479e |
| 124 | >7000000 | Non-inhibitor | C1=CC(=CC=C1CN=C(N)N)C(=O)O | doi:10.1021/jm970479e |
| 125 | >7000000 | Non-inhibitor | CC(=O)NC1=CC=C(C=C1)P(=O)(O)O | doi:10.1021/jm970479e |
| 126 | >7000000 | Non-inhibitor | CC(=O)NC1=CC=C(C=C1)C(=O)N | doi:10.1021/jm970479e |
| 127 | >7000000 | Non-inhibitor | CC(=O)OC1=CC=C(C=C1)C(=O)O | doi:10.1021/jm970479e |
| 128 | >7000000 | Non-inhibitor | CC(=O)NC1=C(C=C(C=C1)C(=O)O)/C=C/CO | doi:10.1021/jm970479e |
| 129 | >7000000 | Non-inhibitor | CC(=O)NC1=C(C=C(C=C1)C(=O)O)CC(CO)O | doi:10.1021/jm970479e |
| 130 | >7000000 | Non-inhibitor | CC(C)C(=O)NC1=CC=C(C=C1)C(=O)O | doi:10.1021/jm970479e |
| 131 | >7000000 | Non-inhibitor | C1=CC(=CC=C1C(=O)O)NC(=O)C(F)(F)F | doi:10.1021/jm970479e |
| 132 | >7000000 | Non-inhibitor | CC(=O)NCCC1=CC=CC(=C1)C(=O)O | doi:10.1021/jm970479e |
| 133 | >7000000 | Non-inhibitor | CC(=O)NC1=C(C=C(C=C1)C(=O)O)CCO | doi:10.1021/jm970479e |
| 134 | >7000000 | Non-inhibitor | C1=CC(=CC(=C1)CCN)C(=O)O | doi:10.1021/jm970479e |
| 135 | >7000000 | Non-inhibitor | C1=CC(=CC(=C1)CC(=N)N)C(=O)O | doi:10.1021/jm970479e |
| 136 | >7000000 | Non-inhibitor | C1=CC(=CC(=C1)CN)C(=O)O | doi:10.1021/jm970479e |
| 137 | >7000000 | Non-inhibitor | CC(=O)NC1=C(C=C(C=C1)C(=O)O)OCCO | doi:10.1021/jm970479e |
| 138 | >7000000 | Non-inhibitor | CC(=O)NC1=C(C=C(C=C1)C(=O)O)CO | doi:10.1021/jm970479e |
| 139 | >7000000 | Non-inhibitor | C1=CC(=CC(=C1)CN=C(N)N)C(=O)O | doi:10.1021/jm970479e |
| 140 | >7000000 | Non-inhibitor | CC(=O)NCC1=CC=CC(=C1)C(=O)O | doi:10.1021/jm970479e |
| 141 | >7000000 | Non-inhibitor | C1=CC(=CC(=C1)N=C(N)NC#N)C(=O)O | doi:10.1021/jm970479e |
| 142 | >7000000 | Non-inhibitor | C1=C(C=C(C=C1N=C(N)N)C(=O)O)CN=C(N)N | doi:10.1021/jm970479e |
| 143 | >7000000 | Non-inhibitor | CN(C)C(=O)NC1=CC=C(C=C1)C(=O)O | doi:10.1021/jm970479e |
| 144 | >7000000 | Non-inhibitor | CC(=O)NCCC1=CC=C(C=C1)C(=O)O | doi:10.1021/jm970479e |
| 145 | >7000000 | Non-inhibitor | C1=CC(=CC=C1CCN)C(=O)O | doi:10.1021/jm970479e |
| 146 | >7000000 | Non-inhibitor | C1=CC(=CC=C1C(=O)O)NS(=O)(=O)C(F)(F)F | doi:10.1021/jm970479e |
| 147 | >7000000 | Non-inhibitor | CS(=O)CC1=CC=C(C=C1)C(=O)O | doi:10.1021/jm970479e |
| 148 | >7000000 | Non-inhibitor | CNC(=O)OC1=CC=C(C=C1)C(=O)O | doi:10.1021/jm970479e |
| 149 | >7000000 | Non-inhibitor | CS(=O)(=O)NC1=CC=C(C=C1)C(=O)O | doi:10.1021/jm970479e |
| 150 | >7000000 | Non-inhibitor | CC(=S)NC1=CC=C(C=C1)C(=O)O | doi:10.1021/jm970479e |
| 151 | >7000000 | Non-inhibitor | CS(=O)(=O)CC1=CC=C(C=C1)C(=O)O | doi:10.1021/jm970479e |
| 152 | >7000000 | Non-inhibitor | C1=C(C=C(C=C1N)C(=O)O)CN=C(N)N | doi:10.1021/jm970479e |
| 153 | >7000000 | Non-inhibitor | CC(=O)NCC1=CC=C(C=C1)C(=O)O | doi:10.1021/jm970479e |
| 154 | >7000000 | Non-inhibitor | C1=CC(=C(C=C1C(=O)O)N=C(N)N)CCO | doi:10.1021/jm970479e |
| 155 | >7000000 | Non-inhibitor | CC(=O)NC1C(C=C(OC1[C@H](C(CO)O)O)C(=O)O)O | doi:10.1021/jm970479e |
| 156 | >7000000 | Non-inhibitor | CC(=O)NC1=C(C=C(C=C1)C(=O)O)CC(=O)N | doi:10.1021/jm970479e |
| 157 | >7000000 | Non-inhibitor | C1=CC(=CC(=C1)NC(=O)N)C(=O)O | doi:10.1021/jm970479e |
| 158 | >7000000 | Non-inhibitor | CS(=O)(=O)NC1=C(C=C(C=C1)C(=O)O)C(CN)O | doi:10.1021/jm970479e |
| 159 | >7000000 | Non-inhibitor | CC(=O)NC1=C(C=C(C=C1)C(=O)O)CCN | doi:10.1021/jm970479e |
| 160 | >7000000 | Non-inhibitor | C1=CC(=CC=C1CCN=C(N)N)C(=O)O | doi:10.1021/jm970479e |
| 161 | >7000000 | Non-inhibitor | C1=C(C=C(C=C1N=C(N)N)C(=O)O)CCN | doi:10.1021/jm970479e |
| 162 | >50000 | Non-inhibitor | CC(=O)NC1=C(C=C(C=C1)C(=O)O)/C=N/N=C(N)N | doi:10.1021/jm970479e |
| 163 | >3500000 | Non-inhibitor | CC(=O)NC1=C(C=C(C=C1)C(=O)O)[N+](=O)[O-] | doi:10.1021/jm970479e |
| 164 | >3500000 | Non-inhibitor | C1=CC(=CC(=C1)NC2N=NN=N2)C(=O)O | doi:10.1021/jm970479e |
| 165 | >900000 | Non-inhibitor | C1=COC(=C1)C(=O)NC2=C(C=C(C=C2)C(=O)O)N=C(N)N | doi:10.1021/jm970479e |
| 166 | >3500000 | Non-inhibitor | CC(=O)NC1=C(C=C(C=C1)C(=O)O)N | doi:10.1021/jm970479e |
| 167 | >7000000 | Non-inhibitor | C1=C(C=C(C=C1NC#N)NC#N)C(=O)O | doi:10.1021/jm970479e |
| 168 | 250000 | Non-inhibitor | C1CC(=O)N(C1)C2=C(C=C(C=C2)C(=O)O)N=C(N)N | doi:10.1016/S0968-0896(99)00197-2 |
| 169 | 8000 | Inhibitor | C(C)(=O)NC1=C(C=C(C(=O)O)C=C1N)OC(CC)CC | doi:10.1016/S0968-0896(99)00197-2 |
| 170 | 1000 | Inhibitor | C(C)(=O)NC1=C(C=C(C(=O)O)C=C1NC(=N)N)OC(CC)CC | doi:10.1016/S0968-0896(99)00197-2 |
| 171 | 5 | Inhibitor | CC(=O)N[C@H]1[C@@H](C=C(O[C@@H]1[C@@H]([C@H](CO)O)O)C(=O)O)N=C(N)N | doi:10.1016/S0968-0896(99)00197-2 |
| 172 | 1 | Inhibitor | CCCN(CCC)C(=O)[C@@H]1[C@H]([C@@H](C=C(O1)C(=O)O)N=C(N)N)NC(=O)C | doi:10.1016/S0968-0896(99)00197-2 |
| 173 | 230000 | Non-inhibitor | C(C)(=O)NC1=C(C=C(C(=O)O)C=C1)OC(CCC)CCC | doi:10.1016/S0968-0896(99)00197-2 |
| 174 | >390000 | Non-inhibitor | CC(=O)N[C@@H]1[C@H](C=C(O[C@H]1C(=O)NCCN)C(=O)O)N | doi:10.1021/jm970374b |
| 175 | 8 | Inhibitor | CC(C)N(CCC1=CC=CC=C1)C(=O)[C@H]2[C@@H]([C@H](C=C(O2)C(=O)O)N)NC(=O)C | doi:10.1021/jm970374b |
| 176 | 46 | Inhibitor | CC(=O)N[C@@H]1[C@H](C=C(O[C@H]1C(=O)N(CCC2=CC=CC=C2)C3CC3)C(=O)O)N | doi:10.1021/jm970374b |
| 177 | 3000 | Inhibitor | CC(=O)N[C@@H]1[C@H](C=C(O[C@H]1C(=O)N(CCCO)CCC2=CC=CC=C2)C(=O)O)N | doi:10.1021/jm970374b |
| 178 | 8 | Inhibitor | CCCN(CCC1=CC=C(C=C1)OC)C(=O)[C@H]2[C@@H]([C@H](C=C(O2)C(=O)O)N)NC(=O)C | doi:10.1021/jm970374b |
| 179 | 9 | Inhibitor | CCCN(CCC1=CC=C(C=C1)O)C(=O)[C@H]2[C@@H]([C@H](C=C(O2)C(=O)O)N)NC(=O)C | doi:10.1021/jm970374b |
| 180 | 5 | Inhibitor | CCCN(CCC1=CC(=CC=C1)OC)C(=O)[C@H]2[C@@H]([C@H](C=C(O2)C(=O)O)N)NC(=O)C | doi:10.1021/jm970374b |
| 181 | 3 | Inhibitor | CCCN(CCC1=CC(=CC=C1)O)C(=O)[C@H]2[C@@H]([C@H](C=C(O2)C(=O)O)N)NC(=O)C | doi:10.1021/jm970374b |
| 182 | 6 | Inhibitor | CCCN(CCC1=C(C=C(C=C1)Cl)Cl)C(=O)[C@H]2[C@@H]([C@H](C=C(O2)C(=O)O)N)NC(=O)C | doi:10.1021/jm970374b |
| 183 | 12 | Inhibitor | CCCN(CCC1=CC=C(C=C1)C(C)C)C(=O)[C@H]2[C@@H]([C@H](C=C(O2)C(=O)O)N)NC(=O)C | doi:10.1021/jm970374b |
| 184 | 270 | Inhibitor | CCCN(CCC1=CC=C(C=C1)CC2=CC=CC=C2)C(=O)[C@H]3[C@@H]([C@H](C=C(O3)C(=O)O)N)NC(=O)C | doi:10.1021/jm970374b |
| 185 | 9 | Inhibitor | CCCN(CCC1=CC=CC=C1OC)C(=O)[C@H]2[C@@H]([C@H](C=C(O2)C(=O)O)N)NC(=O)C | doi:10.1021/jm970374b |
| 186 | 2 | Inhibitor | CCCN(CCC1=CC=CC(=C1)C2=CC=CC=C2)C(=O)[C@H]3[C@@H]([C@H](C=C(O3)C(=O)O)N)NC(=O)C | doi:10.1021/jm970374b |
| 187 | 2900 | Inhibitor | CC(=O)N[C@@H]1[C@H](C=C(O[C@H]1C(=O)N2CCCC2)C(=O)O)N | doi:10.1021/jm970374b |
| 188 | 310 | Inhibitor | CC1CCCN1C(=O)[C@H]2[C@@H]([C@H](C=C(O2)C(=O)O)N)NC(=O)C | doi:10.1021/jm970374b |
| 189 | 5 | Inhibitor | C[C@H]1CC[C@H](N1C(=O)[C@H]2[C@@H]([C@H](C=C(O2)C(=O)O)N)NC(=O)C)C | doi:10.1021/jm970374b |
| 190 | 110 | Inhibitor | C[C@@H]1CC[C@H](N1C(=O)[C@H]2[C@@H]([C@H](C=C(O2)C(=O)O)N)NC(=O)C)C | doi:10.1021/jm970374b |
| 191 | 110 | Inhibitor | C[C@H]1CC[C@@H](N1C(=O)[C@H]2[C@@H]([C@H](C=C(O2)C(=O)O)N)NC(=O)C)C | doi:10.1021/jm970374b |
| 192 | 290 | Inhibitor | CC[C@H]1CC[C@H](N1C(=O)[C@H]2[C@@H]([C@H](C=C(O2)C(=O)O)N)NC(=O)C)CC | doi:10.1021/jm970374b |
| 193 | 420000 | Non-inhibitor | CC(=O)N[C@@H]1[C@H](C=C(O[C@H]1C(=O)NCCO)C(=O)O)N | doi:10.1021/jm970374b |
| 194 | >480000 | Non-inhibitor | CC(=O)N[C@@H]1[C@H](C=C(O[C@H]1C(=O)NCC(=O)O)C(=O)O)N | doi:10.1021/jm970374b |
| 195 | 6 | Inhibitor | CC1CCC(N1C(=O)[C@H]2[C@@H]([C@H](C=C(O2)C(=O)O)N)NC(=O)C)C | doi:10.1021/jm970374b |
| 196 | 40 | Inhibitor | CC(=O)N[C@@H]1[C@H](C=C(O[C@H]1C(=O)N(CC=C)CC=C)C(=O)O)N | doi:10.1021/jm970374b |
| 197 | 4200 | Inhibitor | CCCN(CC1=CC=CC=C1)C(=O)[C@H]2[C@@H]([C@H](C=C(O2)C(=O)O)N)NC(=O)C | doi:10.1021/jm970374b |
| 198 | 18 | Inhibitor | CCCN(CCCC1=CC=CC=C1)C(=O)[C@H]2[C@@H]([C@H](C=C(O2)C(=O)O)N)NC(=O)C | doi:10.1021/jm970374b |
| 199 | 130000 | Non-inhibitor | CC(=O)N[C@@H]1[C@H](C=C(O[C@H]1C(=O)N(CCC2=CC=CC=C2)CC(=O)O)C(=O)O)N | doi:10.1021/jm970374b |
| 200 | >300000 | Non-inhibitor | CC(=O)N[C@@H]1[C@H](C=C(O[C@H]1C(=O)N(CCC2=CC=CC=C2)CCN(C)C)C(=O)O)N | doi:10.1021/jm970374b |
| 201 | 9 | Inhibitor | CC(=O)N[C@@H]1[C@H](C=C(O[C@H]1C(=O)N(CCC2=CC=CC=C2)CCN=[N+]=[N-])C(=O)O)N | doi:10.1021/jm970374b |
| 202 | 12 | Inhibitor | CC(=O)N[C@@H]1[C@H](C=C(O[C@H]1C(=O)N(CCC2=CC=CC=C2)CCO)C(=O)O)N | doi:10.1021/jm970374b |
| 203 | 4 | Inhibitor | CCCN(C)C(=O)[C@H]1[C@@H]([C@H](C=C(O1)C(=O)O)N=C(N)N)NC(=O)C | doi:10.1021/jm970374b |
| 204 | 2 | Inhibitor | CCCN(CCC)C(=O)[C@H]1[C@@H]([C@H](C=C(O1)C(=O)O)N=C(N)N)NC(=O)C | doi:10.1021/jm970374b |
| 205 | 1 | Inhibitor | CCN(CC)C(=O)[C@H]1[C@@H]([C@H](C=C(O1)C(=O)O)N=C(N)N)NC(=O)C | doi:10.1021/jm970374b |
| 206 | 7 | Inhibitor | CCCCCCCCCN(CCC)C(=O)[C@H]1[C@@H]([C@H](C=C(O1)C(=O)O)N=C(N)N)NC(=O)C | doi:10.1021/jm970374b |
| 207 | >100000 | Non-inhibitor | CC(=O)NC1=C(C=C(C=C1N=C(N)N)C(=O)O)[C@@H]([C@@H](CO)O)O | doi:10.1016/0960-894X(95)00390-F |
| 208 | 5026.666667 | Inhibitor | CC(C)C[C@@H]([C@H]1[C@@H](C[C@@H](N1)C(=O)O)N)NC(=O)C | doi:10.1021/jm049276y |
| 209 | 1127.777778 | Inhibitor | CC(C)C[C@@H]([C@H]1[C@@H](C[C@@H](N1)C(=O)O)C2=NC=CN2)NC(=O)C | doi:10.1021/jm049276y |
| 210 | 870 | Inhibitor | CC(C)C[C@@H]([C@H]1[C@@H](C[C@@H](N1)C(=O)O)C2=NOC=C2)NC(=O)C | doi:10.1021/jm049276y |
| 211 | 837.7777778 | Inhibitor | CC(C)C[C@@H]([C@H]1[C@@H](C[C@@H](N1)C(=O)O)C(=O)OC)NC(=O)C | doi:10.1021/jm049276y |
| 212 | 128888.8889 | Non-inhibitor | CC(C)C[C@@H]([C@H]1[C@@H](C[C@@H](N1)C(=O)O)C(=O)NC)NC(=O)C | doi:10.1021/jm049276y |
| 213 | 1965.555556 | Inhibitor | CC(C)C[C@@H]([C@H]1[C@@H](C[C@@H](N1)C(=O)O)C2=CN=CN2)NC(=O)C | doi:10.1021/jm049276y |
| 214 | 1127.777778 | Inhibitor | CC(C)C[C@@H]([C@H]1[C@@H](C[C@@H](N1)C(=O)O)C2=CSC=N2)NC(=O)C | doi:10.1021/jm049276y |
| 215 | 998.8888889 | Inhibitor | CC(C)C[C@@H]([C@H]1[C@@H](C[C@@H](N1)C(=O)O)C2=NC=CS2)NC(=O)C | doi:10.1021/jm049276y |
| 216 | 145 | Inhibitor | CC(C)C[C@@H]([C@H]1[C@@H](C[C@@H](N1)C(=O)O)C2=CC=NN2)NC(=O)C | doi:10.1021/jm049276y |
| 217 | 483.3333333 | Inhibitor | C/C=C/[C@@H]1C[C@@H](N[C@H]1[C@H](CC(C)C)NC(=O)C)C(=O)O | doi:10.1021/jm049276y |
| 218 | 966.6666667 | Inhibitor | CC(C)C[C@@H]([C@H]1[C@@H](C[C@@H](N1)C(=O)O)C=C)NC(=O)C | doi:10.1021/jm049276y |
| 219 | 64.44444444 | Inhibitor | C/C=C\[C@@H]1C[C@@H](N[C@H]1[C@H](CC(C)C)NC(=O)C)C(=O)O | doi:10.1021/jm049276y |
| 220 | 1256.666667 | Inhibitor | CC(C)C[C@@H]([C@H]1[C@@H](C[C@@H](N1)C(=O)O)C=C(C)C)NC(=O)C | doi:10.1021/jm049276y |
| 221 | 1353.333333 | Inhibitor | CC(C)C[C@@H]([C@H]1[C@@H](C[C@@H](N1)C(=O)O)C=C(F)F)NC(=O)C | doi:10.1021/jm049276y |
| 222 | 254.5555556 | Inhibitor | CC(C)C[C@@H]([C@H]1[C@@H](C[C@@H](N1)C(=O)O)/C=C\Cl)NC(=O)C | doi:10.1021/jm049276y |
| 223 | 9666.666667 | Inhibitor | CC(C)C[C@@H]([C@H]1[C@@H](C[C@@H](N1)C(=O)O)/C=C/Cl)NC(=O)C | doi:10.1021/jm049276y |
| 224 | 330 | Inhibitor | CCNC(=O)C([C@H]1CC(C[C@@H]1N=C(N)N)C(=O)O)NC(=O)C | doi:10.1021/jm0303406 |
| 225 | 1340 | Inhibitor | CC(C)NC(=O)C([C@H]1CC(C[C@@H]1N=C(N)N)C(=O)O)NC(=O)C | doi:10.1021/jm0303406 |
| 226 | 720 | Inhibitor | CC(=O)NC([C@H]1CC(C[C@@H]1N=C(N)N)C(=O)O)C(=O)NCC=C | doi:10.1021/jm0303406 |
| 227 | 9900 | Inhibitor | CCCCNC(=O)C([C@H]1CC(C[C@@H]1N=C(N)N)C(=O)O)NC(=O)C | doi:10.1021/jm0303406 |
| 228 | 410 | Inhibitor | CCC(C)NC(=O)C([C@H]1CC(C[C@@H]1N=C(N)N)C(=O)O)NC(=O)C | doi:10.1021/jm0303406 |
| 229 | 90 | Inhibitor | CCC(CC)NC(=O)C([C@H]1CC(C[C@@H]1N=C(N)N)C(=O)O)NC(=O)C | doi:10.1021/jm0303406 |
| 230 | 3200 | Inhibitor | CCCC(C)NC(=O)C([C@H]1CC(C[C@@H]1N=C(N)N)C(=O)O)NC(=O)C | doi:10.1021/jm0303406 |
| 231 | 8400 | Inhibitor | CC(C)CC(C)NC(=O)C([C@H]1CC(C[C@@H]1N=C(N)N)C(=O)O)NC(=O)C | doi:10.1021/jm0303406 |
| 232 | 8700 | Inhibitor | CCCCC(C)NC(=O)C([C@H]1CC(C[C@@H]1N=C(N)N)C(=O)O)NC(=O)C | doi:10.1021/jm0303406 |
| 233 | 1800 | Inhibitor | CCCCC(CC)NC(=O)C([C@H]1CC(C[C@@H]1N=C(N)N)C(=O)O)NC(=O)C | doi:10.1021/jm0303406 |
| 234 | 6400 | Inhibitor | CC(=O)NC([C@H]1CC(C[C@@H]1N=C(N)N)C(=O)O)C(=O)NCCC2=CC=CC=C2 | doi:10.1021/jm0303406 |
| 235 | 230 | Inhibitor | CC(CCC1=CC=CC=C1)NC(=O)C([C@H]2CC(C[C@@H]2N=C(N)N)C(=O)O)NC(=O)C | doi:10.1021/jm0303406 |
| 236 | 4400 | Inhibitor | CC(COC)NC(=O)C([C@H]1CC(C[C@@H]1N=C(N)N)C(=O)O)NC(=O)C | doi:10.1021/jm0303406 |
| 237 | 2200 | Inhibitor | CCC(COC)NC(=O)C([C@H]1CC(C[C@@H]1N=C(N)N)C(=O)O)NC(=O)C | doi:10.1021/jm0303406 |
| 238 | 940 | Inhibitor | CCCN(C)C(=O)C([C@H]1CC(C[C@@H]1N=C(N)N)C(=O)O)NC(=O)C | doi:10.1021/jm0303406 |
| 239 | 3210 | Inhibitor | CC(C)N(C)C(=O)C([C@H]1CC(C[C@@H]1N=C(N)N)C(=O)O)NC(=O)C | doi:10.1021/jm0303406 |
| 240 | 650 | Inhibitor | CC(=O)NC([C@H]1CC(C[C@@H]1N=C(N)N)C(=O)O)C(=O)N(C)CC=C | doi:10.1021/jm0303406 |
| 241 | 2400 | Inhibitor | CCCCN(C)C(=O)C([C@H]1CC(C[C@@H]1N=C(N)N)C(=O)O)NC(=O)C | doi:10.1021/jm0303406 |
| 242 | 8000 | Inhibitor | CC(=O)NC([C@H]1CC(C[C@@H]1N=C(N)N)C(=O)O)C(=O)N(C)CCC2=CC=CC=C2 | doi:10.1021/jm0303406 |
| 243 | 15 | Inhibitor | CCN(CC)C(=O)C([C@H]1CC(C[C@@H]1N=C(N)N)C(=O)O)NC(=O)C | doi:10.1021/jm0303406 |
| 244 | 130 | Inhibitor | CCCN(CC)C(=O)C([C@H]1CC(C[C@@H]1N=C(N)N)C(=O)O)NC(=O)C | doi:10.1021/jm0303406 |
| 245 | 430 | Inhibitor | CCCCN(CC)C(=O)C([C@H]1CC(C[C@@H]1N=C(N)N)C(=O)O)NC(=O)C | doi:10.1021/jm0303406 |
| 246 | 670 | Inhibitor | CCN(CC1=CC=CC=C1)C(=O)C([C@H]2CC(C[C@@H]2N=C(N)N)C(=O)O)NC(=O)C | doi:10.1021/jm0303406 |
| 247 | 720 | Inhibitor | CCN(CCO)C(=O)C([C@H]1CC(C[C@@H]1N=C(N)N)C(=O)O)NC(=O)C | doi:10.1021/jm0303406 |
| 248 | 200 | Inhibitor | CCCN(CC1CC1)C(=O)C([C@H]2CC(C[C@@H]2N=C(N)N)C(=O)O)NC(=O)C | doi:10.1021/jm0303406 |
| 249 | 60 | Inhibitor | CCCN(CCC)C(=O)C([C@H]1CC(C[C@@H]1N=C(N)N)C(=O)O)NC(=O)C | doi:10.1021/jm0303406 |
| 250 | 920 | Inhibitor | CC(=O)NC([C@H]1CC(C[C@@H]1N=C(N)N)C(=O)O)C(=O)N2CCC2 | doi:10.1021/jm0303406 |
| 251 | 470 | Inhibitor | CC(=O)NC([C@H]1CC(C[C@@H]1N=C(N)N)C(=O)O)C(=O)N2CCCC2 | doi:10.1021/jm0303406 |
| 252 | 150 | Inhibitor | CC(=O)NC([C@H]1CC(C[C@@H]1N=C(N)N)C(=O)O)C(=O)N2CCCCC2 | doi:10.1021/jm0303406 |
| 253 | 990 | Inhibitor | CC(=O)NC([C@H]1CC(C[C@@H]1N=C(N)N)C(=O)O)C(=O)N2CCCCCC2 | doi:10.1021/jm0303406 |
| 254 | 240 | Inhibitor | CC1CCCCN1C(=O)C([C@H]2CC(C[C@@H]2N=C(N)N)C(=O)O)NC(=O)C | doi:10.1021/jm0303406 |
| 255 | 180 | Inhibitor | CCC1CCCCN1C(=O)C([C@H]2CC(C[C@@H]2N=C(N)N)C(=O)O)NC(=O)C | doi:10.1021/jm0303406 |
| 256 | 150 | Inhibitor | CCCC1CCCCN1C(=O)C([C@H]2CC(C[C@@H]2N=C(N)N)C(=O)O)NC(=O)C | doi:10.1021/jm0303406 |
| 257 | 2550 | Inhibitor | CC1CCCN(C1)C(=O)C([C@H]2CC(C[C@@H]2N=C(N)N)C(=O)O)NC(=O)C | doi:10.1021/jm0303406 |
| 258 | 8200 | Inhibitor | CC1CCN(CC1)C(=O)C([C@H]2CC(C[C@@H]2N=C(N)N)C(=O)O)NC(=O)C | doi:10.1021/jm0303406 |
| 259 | 3500 | Inhibitor | CC1CC(CN(C1)C(=O)C([C@H]2CC(C[C@@H]2N=C(N)N)C(=O)O)NC(=O)C)C | doi:10.1021/jm0303406 |
| 260 | 40 | Inhibitor | CC(=O)NC([C@H]1CC(C[C@@H]1N=C(N)N)C(=O)O)C(=O)N2CCOCC2 | doi:10.1021/jm0303406 |
| 261 | 230 | Inhibitor | CC(=O)NC([C@H]1CC(C[C@@H]1N=C(N)N)C(=O)O)C(=O)N2CCSCC2 | doi:10.1021/jm0303406 |
| 262 | 5500000 | Non-inhibitor | CC(=O)NC1=C(C=C(C=C1)C(=O)O)/C=N/O | doi:10.1016/j.bmc.2005.01.042 |
| 263 | 400000 | Non-inhibitor | CC(=O)NC1=C(C=C(C=C1N=C(N)N)C(=O)O)CCO | doi:10.1016/j.bmc.2005.01.042 |
| 264 | 100000 | Non-inhibitor | CS(=O)(=O)NC1=C(C=C(C=C1)C(=O)O)N=C(N)N | doi:10.1016/j.bmc.2005.01.042 |
| 265 | 2000000 | Non-inhibitor | CS(=O)(=O)NC1=C(C=C(C=C1)C(=O)O)/C=N/O | doi:10.1016/j.bmc.2005.01.042 |
| 266 | 2000000 | Non-inhibitor | CS(=O)(=O)NC1=C(C=C(C=C1N=C(N)N)C(=O)O)CO | doi:10.1016/j.bmc.2005.01.042 |
| 267 | 80000 | Non-inhibitor | CS(=O)(=O)NC1=C(C=C(C=C1N=C(N)N)C(=O)O)/C=N/O | doi:10.1016/j.bmc.2005.01.042 |
| 268 | >100000 | Non-inhibitor | CC(=O)NC1=C(C=C(C=C1N=C(N)N)C(=O)O)C(C(CO)O)O | doi:10.1016/j.bmc.2005.01.042 |
| 269 | 10000 | Inhibitor | CC(=O)NC1=C(C=C(C=C1N=C(N)N)C(=O)O)N=C(N)N | doi:10.1016/j.bmc.2005.01.042 |
| 270 | 3000 | Inhibitor | CCC(CC)OC1=C(C=C(C=C1N=C(N)N)C(=O)O)N=C(N)N | doi:10.1016/j.bmc.2005.01.042 |
| 271 | 3000000 | Non-inhibitor | CS(=O)(=O)NC1=CN=C(C=C1)C(=O)O | doi:10.1016/j.bmc.2005.01.042 |
| 272 | 2000000 | Non-inhibitor | CC(=O)NC1=CN=C(C=C1)C(=O)O | doi:10.1016/j.bmc.2005.01.042 |
| 273 | 400000 | Non-inhibitor | CC(=S)NC1=CN=C(C=C1)C(=O)O | doi:10.1016/j.bmc.2005.01.042 |
| 274 | 3000000 | Non-inhibitor | CC(=O)NC1=C(N=C(C=C1)C(=O)O)N | doi:10.1016/j.bmc.2005.01.042 |
| 275 | 6000 | Inhibitor | CC(=O)NC1=C(N=C(C=C1)C(=O)O)N=C(N)N | doi:10.1016/j.bmc.2005.01.042 |
| 276 | 70000 | Non-inhibitor | CC(=O)NC1=CN=C(C=C1N)C(=O)O | doi:10.1016/j.bmc.2005.01.042 |
| 277 | 1000000 | Non-inhibitor | CS(=O)(=O)NC1=C(N=C(C=C1N)C(=O)O)N | doi:10.1016/j.bmc.2005.01.042 |
| 278 | 4000 | Inhibitor | CC(=O)NC1=C(N=C(C=C1N)C(=O)O)N=C(N)N | doi:10.1016/j.bmc.2005.01.042 |
| 279 | >7000000 | Non-inhibitor | C1=C(C=C(C(=C1CCO)N)N=C(N)N)C(=O)O | doi:10.1016/j.bmc.2005.01.042 |
| 280 | 50 | Inhibitor | CCCCC([C@@H]1CC(C[C@H]1N=C(N)N)C(=O)O)NC(=O)C | doi:10.1021/jm010277p |
| 281 | 25 | Inhibitor | CCCC[C@@H]([C@@H]1CC(C[C@H]1N=C(N)N)C(=O)O)NC(=O)C | doi:10.1021/jm010277p |
| 282 | 359 | Inhibitor | CCCC[C@@H]([C@@H]1C[C@@H](C[C@H]1N=C(N)N)C(=O)O)NC(=O)C | doi:10.1021/jm010277p |
| 283 | 412 | Inhibitor | CCCC[C@@H]([C@@H]1C[C@H](C[C@H]1N=C(N)N)C(=O)O)NC(=O)C | doi:10.1021/jm010277p |
| 284 | <1 | Inhibitor | CCC(CC)[C@@H]([C@@H]1C[C@H](C[C@H]1N=C(N)N)C(=O)O)NC(=O)C | doi:10.1021/jm010277p |
| 285 | 17 | Inhibitor | CCC(CC)O[C@@H]1C=C(C[C@@H]([C@H]1NC(=O)C)N)C(=O)OCC | doi:10.1016/j.bmcl.2013.04.033 |
| 286 | 7200 | Inhibitor | CCC(CC)C(=O)NC1=C(C=CC(=C1)NC(=O)/C=C/C2=CC(=C(C=C2)O)O)OCC(=O)O | doi:10.1016/j.bmcl.2013.04.033 |
| 287 | >100000 | Non-inhibitor | CC(=O)NC1=C(C=CC(=C1)NC(=O)/C=C/C2=CC=CC=C2)O | doi:10.1016/j.bmcl.2013.04.033 |
| 288 | >100000 | Non-inhibitor | C1CC1C(=O)NC2=C(C=CC(=C2)NC(=O)/C=C/C3=CC=CC=C3)O | doi:10.1016/j.bmcl.2013.04.033 |
| 289 | >100000 | Non-inhibitor | CCC(=O)NC1=C(C=CC(=C1)NC(=O)/C=C/C2=CC=CC=C2)O | doi:10.1016/j.bmcl.2013.04.033 |
| 290 | >100000 | Non-inhibitor | CC(C)C(=O)NC1=C(C=CC(=C1)NC(=O)/C=C/C2=CC=CC=C2)O | doi:10.1016/j.bmcl.2013.04.033 |
| 291 | >100000 | Non-inhibitor | CCC(CC)C(=O)NC1=C(C=CC(=C1)NC(=O)/C=C/C2=CC=CC=C2)O | doi:10.1016/j.bmcl.2013.04.033 |
| 292 | 53132.85874 | Non-inhibitor | CC(C)(C=C)C1=C(C=C(C(=C1)/C=C/C(=O)C2=CC=C(C=C2)O)OC)O | doi:10.1016/j.bmcl.2010.11.016 |
| 293 | 175478.6201 | Non-inhibitor | CC(=CCC1=C(C=CC(=C1)/C=C/C(=O)C2=C(C=C(C=C2)O)O)O)C | doi:10.1016/j.bmcl.2010.11.016 |
| 294 | 104298.2637 | Non-inhibitor | CC(=CCC1=C(C(=CC(=C1)/C=C/C(=O)C2=C(C=C(C=C2)O)O)O)O)C | doi:10.1016/j.bmcl.2010.11.016 |
| 295 | 118821.8829 | Non-inhibitor | CC(C)(C=C)C1=C(C=C(C(=C1)/C=C/C(=O)C2=C(C=C(C=C2)O)O)OC)O | doi:10.1016/j.bmcl.2010.11.016 |
| 296 | 134934.4055 | Non-inhibitor | CC(=CCC1=C(C=CC(=C1)/C=C/C(=O)C2=C(C(=C(C=C2)O)CC=C(C)C)O)O)C | doi:10.1016/j.bmcl.2010.11.016 |
| 297 | 99352.13716 | Non-inhibitor | CC(=CCC1=C(C=CC(=C1)C(=O)/C=C/C2=C(C(=C(C=C2)O)O)OC)O)C | doi:10.1016/j.bmcl.2010.11.016 |
| 298 | 85500 | Non-inhibitor | C1=CC(=C(C=C1C2=C(C(=O)C3=C(C=C(C=C3O2)O)O)O[C@H]4[C@@H]([C@H]([C@H]([C@H](O4)CO)O)O)O)O)O | doi:10.1016/j.bmcl.2014.07.010 |
| 299 | 477100 | Non-inhibitor | C1=CC(=C(C=C1C2=C(C(=O)C3=C(C=C(C=C3O2)O)O)O[C@H]4[C@@H]([C@H]([C@@H]([C@H](O4)CO)O)O)O)O)O | doi:10.1016/j.bmcl.2014.07.010 |
| 300 | 51200 | Non-inhibitor | C1=CC(=C(C=C1C2=CC(=O)C3=C(C=C(C=C3O2)O[C@H]4[C@@H]([C@H]([C@@H]([C@H](O4)CO)O)O)O)O)O)O | doi:10.1016/j.bmcl.2014.07.010 |
| 301 | 1212000 | Non-inhibitor | C[C@H]1[C@@H]([C@H]([C@H]([C@@H](O1)OC2=C(OC3=CC(=CC(=C3C2=O)O)O)C4=CC(=C(C=C4)O)O)O)O)O | doi:10.1016/j.bmcl.2014.07.010 |
| 302 | 95900 | Non-inhibitor | C[C@H]1[C@@H]([C@H]([C@H]([C@@H](O1)OC[C@@H]2[C@H]([C@@H]([C@H]([C@@H](O2)OC3=C(OC4=CC(=CC(=C4C3=O)O)O)C5=CC(=C(C=C5)O)O)O)O)O)O)O)O | doi:10.1016/j.bmcl.2014.07.010 |
| 303 | 101433.2514 | Non-inhibitor | C1=CC2=C(C=C1O)C(=O)C3=C(C=C(C=C3O2)O)O | doi:10.1016/j.bmcl.2012.04.028 |
| 304 | 98378.61525 | Non-inhibitor | C1=CC2=C(C(=C1)O)C(=O)C3=C(O2)C=CC(=C3)O | doi:10.1016/j.bmcl.2012.04.028 |
| 305 | 67747.58983 | Non-inhibitor | C1=CC=C2C(=C1)C(=O)C3=C(C=C(C=C3O2)O)O | doi:10.1016/j.bmcl.2012.04.028 |
| 306 | 99099.23865 | Non-inhibitor | COC1=C2C(=C(C=C1)O)C(=O)C3=C(O2)C=CC(=C3)O | doi:10.1016/j.bmcl.2012.04.028 |
| 307 | >367305.410776006 | Non-inhibitor | COC1=C(C=C2C(=C1OC)OC3=CC=CC=C3C2=O)O | doi:10.1016/j.bmcl.2012.04.028 |
| 308 | >370045.552607526 | Non-inhibitor | COC1=C2C(=CC3=C1OCO3)C(=O)C4=CC=CC=C4O2 | doi:10.1016/j.bmcl.2012.04.028 |
| 309 | >346918.668387383 | Non-inhibitor | COC1=C(C=C2C(=C1OC)C(=O)C3=C(O2)C=C(C=C3)O)O | doi:10.1016/j.bmcl.2012.04.028 |
| 310 | 76134.03587 | Non-inhibitor | C1=CC2=C(C(=C1)O)OC3=CC(=C(C(=C3C2=O)O)O)O | doi:10.1016/j.bmcl.2012.04.028 |
| 311 | >412835.893603934 | Non-inhibitor | COC1=CC=CC2=C1C(=O)C3=C(O2)C=CC(=C3)O | doi:10.1016/j.bmcl.2012.04.028 |
| 312 | >367305.410776006 | Non-inhibitor | COC1=C(C2=C(C(=C1)O)OC3=CC=CC=C3C2=O)OC | doi:10.1016/j.bmcl.2012.04.028 |
| 313 | >147774.224628643 | Non-inhibitor | CC(=CCC1=C(C=C2C(=C1O)C(=O)C(=CO2)C3=CC=C(C=C3)O)O)C | doi:10.1016/j.bmcl.2010.09.077 |
| 314 | 62575.32097 | Non-inhibitor | CC(=CCC1=CC(=CC(=C1O)CC=C(C)C)/C=C/C(=O)C2=C(C=C(C=C2)O)O)C | doi:10.1016/j.bmcl.2010.09.077 |
| 315 | >147774.224628643 | Non-inhibitor | CC(=CCC1=C(C=CC(=C1)C2=COC3=CC(=CC(=C3C2=O)O)O)O)C | doi:10.1016/j.bmcl.2010.09.077 |
| 316 | >156084.647826209 | Non-inhibitor | CC1(C=CC2=C(O1)C=CC(=C2)C3=COC4=C(C3=O)C=CC(=C4)O)C | doi:10.1016/j.bmcl.2010.09.077 |
| 317 | >118351.598930102 | Non-inhibitor | CC(=C)C(CC1=C2C(=C(C(=CO2)C3=CC=C(C=C3)O)O)C(=O)C4C1OC(C=C4)(C)C)O | doi:10.1016/j.bmcl.2010.09.077 |
| 318 | >136476.291338669 | Non-inhibitor | CC1(C=CC2=C(C3=C(C(=C2O1)CO)OC=C(C3=O)C4=CC=C(C=C4)O)O)C | doi:10.1016/j.bmcl.2010.09.077 |
| 319 | >670000 | Non-inhibitor | CCC(CC)O[C@@H]1C=C([C@@H]2CC[C@]1([C@H]2O)C(=O)O)C(=O)O | doi:10.1016/S0960-894X(99)00032-3 |
| 320 | 80000 | Non-inhibitor | CCC(CC)O[C@@H]1C=C([C@@H]2CC[C@]1([C@H]2O)NC(=O)C)C(=O)O | doi:10.1016/S0960-894X(99)00032-3 |
| 321 | 99000 | Non-inhibitor | CCC(CC)O[C@@H]1C=C([C@@H]2CC[C@]1([C@H]2O)C(=O)OC)C(=O)O | doi:10.1016/S0960-894X(99)00032-3 |
| 322 | 89800 | Non-inhibitor | CCC(CC)C(=O)NC1=C(C=CC(=C1)NC(=O)NCC#C)OCC(=O)O | doi:10.1016/j.bmc.2013.10.020 |
| 323 | 83200 | Non-inhibitor | CCC(CC)C(=O)NC1=C(C=C(C(=C1)NC(=O)C(C)C)N=C(N)N)OCC(=O)O | doi:10.1016/j.bmc.2013.10.020 |
| 324 | 3200 | Inhibitor | CCC(CC)C(=O)NC1=C(C=C(C(=C1)NC(=O)C)NC(=O)/C=C/C2=CC(=C(C=C2)O)O)OCC(=O)O | doi:10.1016/j.bmc.2013.10.020 |
| 325 | 9400 | Inhibitor | CCC(CC)C(=O)NC1=C(C=CC(=C1)NC(=O)[C@H](C(C)C)NC(=O)/C=C/C2=CC(=C(C=C2)O)O)OCC(=O)O | doi:10.1016/j.bmc.2013.10.020 |
| 326 | 5800 | Inhibitor | CCC(CC)C(=O)NC1=C(C=C(C(=C1)NC(=O)C(C)C)NC(=O)/C=C/C2=CC(=C(C=C2)O)O)OCC(=O)O | doi:10.1016/j.bmc.2013.10.020 |
| 327 | 8500 | Inhibitor | CCC(CC)C(=O)NC1=C(C=CC(=C1)NC(=O)[C@H](CC(C)C)NC(=O)/C=C/C2=CC(=C(C=C2)O)O)OCC(=O)O | doi:10.1016/j.bmc.2013.10.020 |
| 328 | 100000 | Non-inhibitor | CCC(CC)C(=O)NC1=CC(=C(C=C1)OCC(=O)O)NC(=O)C(CC)CC | doi:10.1016/j.bmc.2013.10.020 |
| 329 | 7900 | Inhibitor | CCC(CC)C(=O)NC1=C(C=CC(=C1)NC(=O)[C@H](C)NC(=O)/C=C/C2=CC(=C(C=C2)O)O)OCC(=O)O | doi:10.1016/j.bmc.2013.10.020 |
| 330 | 69100 | Non-inhibitor | CCC(CC)C(=O)NC1=C(C=C(C(=C1)NC(=O)C)N=C(N)N)OCC(=O)O | doi:10.1016/j.bmc.2013.10.020 |
| 331 | >100000 | Non-inhibitor | CCC(CC)C(=O)NC1=C(C=CC(=C1)NC(=O)C2=CC=CO2)OCC(=O)O | doi:10.1016/j.bmc.2013.10.020 |
| 332 | 9800 | Inhibitor | CCC(CC)C(=O)NC1=C(C=CC(=C1)NC(=O)[C@H](CC2=CC=CC=C2)NC(=O)/C=C/C3=CC(=C(C=C3)O)O)OCC(=O)O | doi:10.1016/j.bmc.2013.10.020 |
| 333 | >100000 | Non-inhibitor | CCC(CC)C(=O)NC1=C(C=CC(=C1)N=C(N)N)OCC(=O)O | doi:10.1016/j.bmc.2013.10.020 |
| 334 | 78600 | Non-inhibitor | CCC(CC)C(=O)NC1=C(C=CC(=C1)NS(=O)(=O)C2=CC=C(C=C2)C)OCC(=O)O | doi:10.1016/j.bmc.2013.10.020 |
| 335 | >100000 | Non-inhibitor | CC[C@H](CO)NC(=O)NC1=CC(=C(C=C1)OCC(=O)O)NC(=O)C(CC)CC | doi:10.1016/j.bmc.2013.10.020 |
| 336 | 9200 | Inhibitor | CCC(CC)C(=O)NC1=C(C=CC(=C1)NC(=O)CNC(=O)/C=C/C2=CC(=C(C=C2)O)O)OCC(=O)O | doi:10.1016/j.bmc.2013.10.020 |
| 337 | >100000 | Non-inhibitor | CCC(CC)C(=O)NC1=C(C=CC(=C1)N)OCC(=O)O | doi:10.1016/j.bmc.2013.10.020 |
| 338 | 54300 | Non-inhibitor | CCC(CC)C(=O)NC1=C(C=C(C(=C1)NC(=O)C(C)C)N)OCC(=O)O | doi:10.1016/j.bmc.2013.10.020 |
| 339 | 4512 | Inhibitor | CCC(CC)O[C@@H]1C=C(C[C@@H]([C@H]1NC(=O)/C=C/C)N)C(=O)O | doi:10.1016/j.bmc.2012.01.026 |
| 340 | 28.1 | Inhibitor | CCC(CC)O[C@@H]1C=C(C[C@@H]([C@H]1NC(=O)C=C)NCC(C)C)C(=O)O | doi:10.1016/j.bmc.2012.01.026 |
| 341 | 99295 | Non-inhibitor | CCC(CC)O[C@@H]1C=C(C[C@@H]([C@H]1NC(=O)/C=C/C)N=[N+]=[N-])C(=O)O | doi:10.1016/j.bmc.2012.01.026 |
| 342 | 2.2 | Inhibitor | CCC(CC)O[C@@H]1C=C(C[C@@H]([C@H]1NC(=O)C)N=[N+]=[N-])C(=O)O | doi:10.1016/j.bmc.2012.01.026 |
| 343 | 0.1 | Inhibitor | CCC(CC)O[C@@H]1C=C(C[C@@H]([C@H]1NC(=O)C=C)N)C(=O)O | doi:10.1016/j.bmc.2012.01.026 |
| 344 | 990 | Inhibitor | CC(=O)NC1=CC(=C(C=C1)C(=O)OC)O | doi:10.1016/j.bmc.2008.01.036 |
| 345 | 220 | Inhibitor | CCOC1=C(C=CC(=C1)NC(=O)C)C(=O)OC | doi:10.1016/j.bmc.2008.01.036 |
| 346 | 970 | Inhibitor | CC(C)CCOC1=CC(=C(C=C1C(=O)OC)N)NC(=O)C | doi:10.1016/j.bmc.2008.01.036 |
| 347 | 41 | Inhibitor | CC(C)CCOC1=CC(=C(C=C1C(=O)O)N=C(N)N)NC(=O)C | doi:10.1016/j.bmc.2008.01.036 |
| 348 | 2690 | Inhibitor | CCCCCCCCCCCCCCCCOC1=C(C=CC(=C1)NC(=O)C)C(=O)OC | doi:10.1016/j.bmc.2008.01.036 |
| 349 | 1540 | Inhibitor | CCCCCCCCCCCCCCCCOC1=CC(=C(C=C1C(=O)OC)[N+](=O)[O-])NC(=O)C | doi:10.1016/j.bmc.2008.01.036 |
| 350 | 590 | Inhibitor | CCCCCCCCCCCCCCCCOC1=CC(=C(C=C1C(=O)OC)N)NC(=O)C | doi:10.1016/j.bmc.2008.01.036 |
| 351 | 2150 | Inhibitor | CCOC1=C(C(=C(C=C1C(=O)OC)[N+](=O)[O-])NC(=O)C)[N+](=O)[O-] | doi:10.1016/j.bmc.2008.01.036 |
| 352 | 310 | Inhibitor | CCOC1=C(C(=C(C=C1C(=O)OC)N)NC(=O)C)N | doi:10.1016/j.bmc.2008.01.036 |
| 353 | 36 | Inhibitor | CCOC1=C(C(=C(C=C1C(=O)OC)N=C(N)N)NC(=O)C)N=C(N)N | doi:10.1016/j.bmc.2008.01.036 |
| 354 | 32 | Inhibitor | CCOC1=C(C(=C(C=C1C(=O)O)N=C(N)N)NC(=O)C)N=C(N)N | doi:10.1016/j.bmc.2008.01.036 |
| 355 | 5320 | Inhibitor | CC(C)OC1=C(C=CC(=C1)NC(=O)C)C(=O)OC | doi:10.1016/j.bmc.2008.01.036 |
| 356 | 570 | Inhibitor | CC(C)OC1=CC(=C(C=C1C(=O)OC)[N+](=O)[O-])NC(=O)C | doi:10.1016/j.bmc.2008.01.036 |
| 357 | 230 | Inhibitor | CC(C)OC1=CC(=C(C=C1C(=O)OC)N)NC(=O)C | doi:10.1016/j.bmc.2008.01.036 |
| 358 | 120 | Inhibitor | CC(C)OC1=CC(=C(C=C1C(=O)OC)N=C(N)N)NC(=O)C | doi:10.1016/j.bmc.2008.01.036 |
| 359 | 49 | Inhibitor | CC(C)OC1=CC(=C(C=C1C(=O)O)N=C(N)N)NC(=O)C | doi:10.1016/j.bmc.2008.01.036 |
| 360 | 9260 | Inhibitor | CCCOC1=C(C=CC(=C1)NC(=O)C)C(=O)OC | doi:10.1016/j.bmc.2008.01.036 |
| 361 | 3570 | Inhibitor | CCCOC1=CC(=C(C=C1C(=O)OC)[N+](=O)[O-])NC(=O)C | doi:10.1016/j.bmc.2008.01.036 |
| 362 | 1260 | Inhibitor | CCCOC1=CC(=C(C=C1C(=O)OC)N)NC(=O)C | doi:10.1016/j.bmc.2008.01.036 |
| 363 | 720 | Inhibitor | CCCOC1=CC(=C(C=C1C(=O)O)N=C(N)N)NC(=O)C | doi:10.1016/j.bmc.2008.01.036 |
| 364 | 2970 | Inhibitor | CCC(C)OC1=C(C=CC(=C1)NC(=O)C)C(=O)OC | doi:10.1016/j.bmc.2008.01.036 |
| 365 | 1590 | Inhibitor | CCC(C)OC1=CC(=C(C=C1C(=O)OC)[N+](=O)[O-])NC(=O)C | doi:10.1016/j.bmc.2008.01.036 |
| 366 | 950 | Inhibitor | CCC(C)OC1=CC(=C(C=C1C(=O)OC)N)NC(=O)C | doi:10.1016/j.bmc.2008.01.036 |
| 367 | 740 | Inhibitor | CCC(C)OC1=CC(=C(C=C1C(=O)OC)N=C(N)N)NC(=O)C | doi:10.1016/j.bmc.2008.01.036 |
| 368 | 230 | Inhibitor | CCCCOC1=C(C=CC(=C1)NC(=O)C)C(=O)OC | doi:10.1016/j.bmc.2008.01.036 |
| 369 | 140 | Inhibitor | CCCCOC1=CC(=C(C=C1C(=O)OC)[N+](=O)[O-])NC(=O)C | doi:10.1016/j.bmc.2008.01.036 |
| 370 | 70 | Inhibitor | CCCCOC1=CC(=C(C=C1C(=O)OC)N)NC(=O)C | doi:10.1016/j.bmc.2008.01.036 |
| 371 | 40 | Inhibitor | CCCCOC1=CC(=C(C=C1C(=O)O)N=C(N)N)NC(=O)C | doi:10.1016/j.bmc.2008.01.036 |
| 372 | 490 | Inhibitor | CC(=O)NC1=CC(=C(C=C1)C(=O)OC)OC2CCCC2 | doi:10.1016/j.bmc.2008.01.036 |
| 373 | 330 | Inhibitor | CC(=O)NC1=C(C=C(C(=C1)OC2CCCC2)C(=O)OC)[N+](=O)[O-] | doi:10.1016/j.bmc.2008.01.036 |
| 374 | 38 | Inhibitor | CC(=O)NC1=C(C=C(C(=C1)OC2CCCC2)C(=O)OC)N | doi:10.1016/j.bmc.2008.01.036 |
| 375 | 3810 | Inhibitor | CC(C)CCOC1=C(C=CC(=C1)NC(=O)C)C(=O)OC | doi:10.1016/j.bmc.2008.01.036 |
| 376 | 2960 | Inhibitor | CC(C)CCOC1=CC(=C(C=C1C(=O)OC)[N+](=O)[O-])NC(=O)C | doi:10.1016/j.bmc.2008.01.036 |
| 377 | 3870 | Inhibitor | CC(C)C[C@@H](C(=O)N1C[C@@H](C[C@H]1C(=O)NO)O)NC(=O)OC(C)(C)C | doi:10.1016/j.bmc.2007.01.020 |
| 378 | 6130 | Inhibitor | CC(C)C[C@@H](C(=O)N1C[C@H](C[C@H]1C(=O)NO)N)NC(=O)OC(C)(C)C | doi:10.1016/j.bmc.2007.01.020 |
| 379 | 2850 | Inhibitor | CC(C)(C)OC(=O)N[C@@H](CC1=CC=CC=C1)C(=O)N2C[C@@H](C[C@H]2C(=O)O)O | doi:10.1016/j.bmc.2007.01.020 |
| 380 | 4270 | Inhibitor | CC(C)(C)OC(=O)N[C@@H](CC1=CC=CC=C1)C(=O)N2C[C@@H](C[C@H]2C(=O)NO)O | doi:10.1016/j.bmc.2007.01.020 |
| 381 | 2400 | Inhibitor | CC(C)(C)OC(=O)N[C@@H](CC1=CC=CC=C1)C(=O)N2C[C@H](C[C@H]2C(=O)O)N | doi:10.1016/j.bmc.2007.01.020 |
| 382 | 4630 | Inhibitor | CC(C)[C@@H](C(=O)N1C[C@@H](C[C@H]1C(=O)O)O)NC(=O)OC(C)(C)C | doi:10.1016/j.bmc.2007.01.020 |
| 383 | 6090 | Inhibitor | CC(C)[C@@H](C(=O)N1C[C@@H](C[C@H]1C(=O)NO)O)NC(=O)OC(C)(C)C | doi:10.1016/j.bmc.2007.01.020 |
| 384 | 4290 | Inhibitor | CC(C)[C@@H](C(=O)N1C[C@H](C[C@H]1C(=O)O)N)NC(=O)OC(C)(C)C | doi:10.1016/j.bmc.2007.01.020 |
| 385 | 90790 | Non-inhibitor | C[C@@H](C(=O)N1C[C@@H](C[C@H]1C(=O)OC)O)NC(=O)OC(C)(C)C | doi:10.1016/j.bmc.2007.01.020 |
| 386 | 83690 | Non-inhibitor | C[C@@H](C(=O)N1C[C@H](C[C@H]1C(=O)OC)N)NC(=O)OC(C)(C)C | doi:10.1016/j.bmc.2007.01.020 |
| 387 | 2150 | Inhibitor | CC[C@H](C)[C@@H](C(=O)N1C[C@@H](C[C@H]1C(=O)NO)O)NC(=O)OC(C)(C)C | doi:10.1016/j.bmc.2007.01.020 |
| 388 | 2710 | Inhibitor | CC[C@H](C)[C@@H](C(=O)N1C[C@H](C[C@H]1C(=O)O)N)NC(=O)OC(C)(C)C | doi:10.1016/j.bmc.2007.01.020 |
| 389 | 2100 | Inhibitor | CC[C@H](C)[C@@H](C(=O)N1C[C@H](C[C@H]1C(=O)NO)N)NC(=O)OC(C)(C)C | doi:10.1016/j.bmc.2007.01.020 |
| 390 | 1560 | Inhibitor | CC(C)(C)OC(=O)N[C@@H](CCSC)C(=O)N1C[C@H](C[C@H]1C(=O)O)N | doi:10.1016/j.bmc.2007.01.020 |
| 391 | 8540 | Inhibitor | C1[C@H](CN([C@@H]1C(=O)NO)C(=O)CC2=CC=CC=C2NC3=C(C=CC=C3Cl)Cl)O | doi:10.1016/j.bmc.2007.01.020 |
| 392 | 3570 | Inhibitor | C1[C@@H](CN([C@@H]1C(=O)NO)C(=O)CC2=CC=CC=C2NC3=C(C=CC=C3Cl)Cl)N | doi:10.1016/j.bmc.2007.01.020 |
| 393 | 4170 | Inhibitor | CC(C)(C)OC(=O)N[C@@H](CC1=CC=CC=C1)C(=O)N2C[C@H](C[C@H]2C(=O)NO)N | doi:10.1016/j.bmc.2007.01.020 |
| 394 | 134400 | Non-inhibitor | C1=CC(=CC=C1C2=COC3=CC(=CC(=C3C2=O)O)O)O | doi:10.1016/j.bmc.2008.06.049 |
| 395 | 87600 | Non-inhibitor | C1=CC(=C(C=C1C2=C(C(=O)C3=C(C=C(C=C3O2)O)O)O)O)O | doi:10.1016/j.bmc.2008.06.049 |
| 396 | >100000 | Non-inhibitor | C1[C@H](OC2=CC(=CC(=C2C1=O)O)O)C3=CC=C(C=C3)O | doi:10.1016/j.bmc.2008.06.049 |
| 397 | >100000 | Non-inhibitor | C1[C@@H]([C@@H](OC2=CC(=CC(=C21)O)O)C3=CC(=C(C=C3)O)O)O | doi:10.1016/j.bmc.2008.06.049 |
| 398 | >100000 | Non-inhibitor | C1C(C(OC2=CC(=CC(=C21)O)O)C3=CC(=C(C=C3)O)O)O | doi:10.1016/j.bmc.2008.06.049 |
| 399 | >100000 | Non-inhibitor | COC1=CC=C(C=C1)C2=COC3=C(C2=O)C=CC(=C3)O | doi:10.1016/j.bmc.2008.06.049 |
| 400 | 83900 | Non-inhibitor | COC1=CC(=C2C(=C1)OC(=C(C2=O)O)C3=CC=C(C=C3)O)O | doi:10.1016/j.bmc.2008.06.049 |
| 401 | >100000 | Non-inhibitor | C[C@H]1[C@@H]([C@H]([C@H]([C@@H](O1)OC[C@@H]2[C@H]([C@@H]([C@H]([C@@H](O2)OC3=CC(=C4C(=O)C[C@H](OC4=C3)C5=CC(=C(C=C5)OC)O)O)O)O)O)O)O)O | doi:10.1016/j.bmc.2008.06.049 |
| 402 | >100000 | Non-inhibitor | C1[C@H](OC2=C(C1=O)C=CC(=C2)O)C3=CC=C(C=C3)O[C@H]4[C@@H]([C@H]([C@@H]([C@H](O4)CO)O)O)O | doi:10.1016/j.bmc.2008.06.049 |
| 403 | >100000 | Non-inhibitor | C1=CC(=CC=C1C2=COC3=CC(=CC(=C3C2=O)O)O)O[C@H]4[C@@H]([C@H]([C@@H]([C@H](O4)CO)O)O)O | doi:10.1016/j.bmc.2008.06.049 |
| 404 | 73300 | Non-inhibitor | C1=CC=C(C=C1)/C=C/2\C(=O)C3=C(O2)C=C(C=C3)O | doi:10.1016/j.bmc.2008.06.049 |
| 405 | >100000 | Non-inhibitor | C1=C(C=C(C(=C1O)O)O)C(=O)O | doi:10.1016/j.bmc.2014.02.014 |
| 406 | >100000 | Non-inhibitor | C1=C2C(=CC(=C1O)O)OC3=CC(=C(C(=C3C2=O)O)O[C@H]4[C@@H]([C@H]([C@@H]([C@H](O4)CO)O)O)O)O | doi:10.1016/j.bmc.2014.02.014 |
| 407 | >100000 | Non-inhibitor | C1=C(C=C(C(=C1O)O)O)C(=O)O[C@H]2[C@@H]([C@H]([C@@H]([C@H](O2)CO)O)O)O | doi:10.1016/j.bmc.2014.02.014 |
| 408 | >100000 | Non-inhibitor | COC1=CC(=C(C(=C1)O[C@H]2[C@@H]([C@H]([C@@H]([C@H](O2)CO)O)O)O)C(=O)C3=CC=C(C=C3)O)O | doi:10.1016/j.bmc.2014.02.014 |
| 409 | >100000 | Non-inhibitor | COC1=C(C(=C(C(=C1)O)C(=O)C2=CC=C(C=C2)O)O)[C@H]3[C@@H]([C@H]([C@@H]([C@H](O3)CO)O)O)O | doi:10.1016/j.bmc.2014.02.014 |
| 410 | >100000 | Non-inhibitor | COC1=C(C(=C2C(=C1)OC3=CC(=C(C=C3C2=O)O)O)O)[C@H]4[C@@H]([C@H]([C@@H]([C@H](O4)CO)O)O)O | doi:10.1016/j.bmc.2014.02.014 |
| 411 | >100000 | Non-inhibitor | C1[C@H]([C@H]([C@@H](C[C@@]1(C(=O)O)O)OC(=O)C2=CC(=C(C(=C2)O)O)O)O)O | doi:10.1016/j.bmc.2014.02.014 |
| 412 | >100000 | Non-inhibitor | C1=CC(=C(C=C1C(=O)OC(=O)C2=CC(=C(C=C2)O)O)O)O | doi:10.1016/j.bmc.2014.02.014 |
| 413 | 850 | Inhibitor | CC1=CC=C(C=C1)CNC(=O)C2=C[C@@H]([C@H]([C@@H](O2)[C@@H]([C@@H](CO)O)O)NC(=O)C)N=C(N)N | doi:10.1021/jm3009713 |
| 414 | 2250 | Inhibitor | CC(C)C[C@@H](C(=O)O)NC(=O)C1=C[C@@H]([C@H]([C@@H](O1)[C@@H]([C@@H](CO)O)O)NC(=O)C)N=C(N)N | doi:10.1021/jm3009713 |
| 415 | 500 | Inhibitor | CC(=O)N[C@@H]1[C@H](C=C(O[C@H]1[C@@H]([C@@H](CO)O)O)C(=O)NCCC(=O)O)N=C(N)N | doi:10.1021/jm3009713 |
| 416 | 917850 | Non-inhibitor | CC[C@H](C)[C@@H](C(=O)O)NC(=O)C1=C[C@@H]([C@H]([C@@H](O1)[C@@H]([C@@H](CO)O)O)NC(=O)C)O | doi:10.1021/jm3009713 |
| 417 | 52950 | Non-inhibitor | CC(=O)N[C@@H]1[C@H](C=C(O[C@H]1[C@@H]([C@@H](CO)O)O)C(=O)N2CCOCC2)O | doi:10.1021/jm3009713 |
| 418 | 5150 | Inhibitor | CC(C)[C@@H](C(=O)O)NC(=O)C1=C[C@@H]([C@H]([C@@H](O1)[C@@H]([C@@H](CO)O)O)NC(=O)C)N | doi:10.1021/jm3009713 |
| 419 | 13 | Inhibitor | CC(=O)N[C@@H]1[C@H](C=C(O[C@H]1[C@@H]([C@@H](CO)O)O)C(=O)NCC2=CC(=CC=C2)F)N=C(N)N | doi:10.1021/jm3009713 |
| 420 | 890 | Inhibitor | CC(=O)N[C@@H]1[C@H](C=C(O[C@H]1[C@@H]([C@@H](CO)O)O)C(=O)NCC2=CC=CC=C2F)N=C(N)N | doi:10.1021/jm3009713 |
| 421 | 857740 | Non-inhibitor | CC(=O)N[C@@H]1[C@H](C=C(O[C@H]1[C@@H]([C@@H](CO)O)O)C(=O)NCC2=CC=C(C=C2)OC)O | doi:10.1021/jm3009713 |
| 422 | 917850 | Non-inhibitor | CC(C)C[C@@H](C(=O)O)NC(=O)C1=C[C@@H]([C@H]([C@@H](O1)[C@@H]([C@@H](CO)O)O)NC(=O)C)O | doi:10.1021/jm3009713 |
| 423 | 60030 | Non-inhibitor | CC(=O)N[C@@H]1[C@H](C=C(O[C@H]1[C@@H]([C@@H](CO)O)O)C(=O)N[C@@H](C2=CC=CC=C2)C(=O)O)O | doi:10.1021/jm3009713 |
| 424 | 107 | Inhibitor | CC(C)[C@@H](C(=O)O)NC(=O)C1=C[C@@H]([C@H]([C@@H](O1)[C@@H]([C@@H](CO)O)O)NC(=O)C)N=C(N)N | doi:10.1021/jm3009713 |
| 425 | 810 | Inhibitor | CC(=O)N[C@@H]1[C@H](C=C(O[C@H]1[C@@H]([C@@H](CO)O)O)C(=O)N[C@@H](CCC(=O)O)C(=O)O)N=C(N)N | doi:10.1021/jm3009713 |
| 426 | 5860 | Inhibitor | CC(=O)N[C@@H]1[C@H](C=C(O[C@H]1[C@@H]([C@@H](CO)O)O)C(=O)N2CCC(CC2)C(=O)O)N | doi:10.1021/jm3009713 |
| 427 | 410 | Inhibitor | C[C@@H](C(=O)O)NC(=O)C1=C[C@@H]([C@H]([C@@H](O1)[C@@H]([C@@H](CO)O)O)NC(=O)C)N=C(N)N | doi:10.1021/jm3009713 |
| 428 | 707140 | Non-inhibitor | CC(=O)N[C@@H]1[C@H](C=C(O[C@H]1[C@@H]([C@@H](CO)O)O)C(=O)NCC2=CC=CC=C2)O | doi:10.1021/jm3009713 |
| 429 | 146320 | Non-inhibitor | CC(=O)N[C@@H]1[C@H](C=C(O[C@H]1[C@@H]([C@@H](CO)O)O)C(=O)N2CCC[C@H](C2)C(=O)O)N | doi:10.1021/jm3009713 |
| 430 | 688380 | Non-inhibitor | CC(C)[C@@H](C(=O)O)NC(=O)C1=C[C@@H]([C@H]([C@@H](O1)[C@@H]([C@@H](CO)O)O)NC(=O)C)O | doi:10.1021/jm3009713 |
| 431 | 646690 | Non-inhibitor | CC(C)C[C@@H](C(=O)O)NC(=O)C1=C[C@@H]([C@H]([C@@H](O1)[C@@H]([C@@H](CO)O)O)NC(=O)C)N | doi:10.1021/jm3009713 |
| 432 | 0.83 | Inhibitor | CCC(CC)O[C@@H]1C=C(C[C@@H]([C@H]1NC(=O)C)N=C(N)N)C(=O)O | doi:10.1021/jm401492x |
| 433 | 1.87 | Inhibitor | CCC(CC)O[C@@H]1C=C(C[C@@H]([C@H]1NC(=O)C)N=C(C)N)C(=O)O | doi:10.1021/jm401492x |
| 434 | 67600 | Non-inhibitor | N(C(=N)N)[C@H]1C[C@H](N(C1)S(=O)(=O)C1=CC=C(C)C=C1)C(=O)O | doi:10.1007/s00044-014-1157-z |
| 435 | 87200 | Non-inhibitor | N(C(=N)N)[C@H]1C[C@H](N(C1)S(=O)(=O)C1=CC=C(C=C1)OC)C(=O)O | doi:10.1007/s00044-014-1157-z |
| 436 | 60700 | Non-inhibitor | C(C1=CC=CC=C1)(=O)N1[C@@H](C[C@@H](C1)NC(=N)N)C(=O)O | doi:10.1007/s00044-014-1157-z |
| 437 | >100000 | Non-inhibitor | C(N)(=N)N1[C@@H](C[C@@H](C1)NC(=O)C=1OC=CC1)C(=O)O | doi:10.1007/s00044-014-1157-z |
| 438 | >100000 | Non-inhibitor | C(C1=CC=CC=C1)(=O)N[C@H]1C[C@H](N(C1)C(N)=N)C(=O)O | doi:10.1007/s00044-014-1157-z |
| 439 | 84000 | Non-inhibitor | C(C=C)OC1=C(C(=O)N[C@H]2C[C@H](N(C2)C(N)=N)C(=O)O)C=CC=C1 | doi:10.1007/s00044-014-1157-z |
| 440 | >100000 | Non-inhibitor | C(N)(=N)N1[C@@H](C[C@@H](C1)NC(C(CC)CC)=O)C(=O)O | doi:10.1007/s00044-014-1157-z |
| 441 | 66600 | Non-inhibitor | C(N)(=N)N1[C@@H](C[C@@H](C1)N1N=NC(=C1)C=1SC=CC1)C(=O)O | doi:10.1007/s00044-014-1157-z |
| 442 | 58700 | Non-inhibitor | C(N)(=N)N1[C@@H](C[C@@H](C1)N1N=NC(=C1)C1=CC=CC=C1)C(=O)O | doi:10.1007/s00044-014-1157-z |
| 443 | 64843.17153 | Non-inhibitor | C1=CC(=CC=C1/C=C/C2=CC(=CC(=C2)O)O)O | doi:10.1055/s-0030-1250030 |
| 444 | 63499.68444 | Non-inhibitor | COC1=C(C=CC(=C1)/C=C/C2=CC(=CC(=C2)O)O)O | doi:10.1055/s-0030-1250030 |
| 445 | 50966.96593 | Non-inhibitor | COC(=O)[C@@H]1[C@H]([C@@H]([C@H]([C@@H](O1)OC2=C(C=CC(=C2)C3=CC(=O)C4=C(C=C(C=C4O3)O)O)O)O)O)O | doi:10.1055/s-2008-1074558 |
| 446 | 16.8 | Inhibitor | CC(=O)N[C@@H]1[C@H](C=C(O[C@H]1[C@@H]([C@@H](CO)O)OC)C(=O)O)N=C(N)N | doi:10.1128/AAC.00333-08 |
| 447 | 164 | Inhibitor | CCCCCCCC(=O)OC[C@H]([C@H]([C@H]1C([C@H](C=C(O1)C(=O)O)N=C(N)N)NC(=O)C)OC)O | doi:10.1128/AAC.00333-08 |
| 448 | 776700 | Non-inhibitor | CC1=CC=C(C=C1)NC(=O)N[C@H]2C=C(O[C@H]([C@@H]2NC(=O)C)[C@@H]([C@@H](CO)O)O)C(=O)O | doi:10.1016/j.ejmech.2012.06.033 |
| 449 | 6600 | Inhibitor | CC(=O)N[C@@H]1[C@H](C=C(O[C@H]1[C@@H]([C@@H](CO)O)O)C(=O)O)NC(=O)NCC2=CC=CO2 | doi:10.1016/j.ejmech.2012.06.033 |
| 450 | 89600 | Non-inhibitor | CC(=O)N[C@@H]1[C@H](C=C(O[C@H]1[C@@H]([C@@H](CO)O)O)C(=O)O)NC(=S)NC2=CC=CC=C2OC | doi:10.1016/j.ejmech.2012.06.033 |
| 451 | 628800 | Non-inhibitor | CC(=O)N[C@@H]1[C@H](C=C(O[C@H]1[C@@H]([C@@H](CO)O)O)C(=O)O)NC(=O)N[C@@H](CC2=CC=CC=C2)C(=O)O | doi:10.1016/j.ejmech.2012.06.033 |
| 452 | 836300 | Non-inhibitor | CC(=O)N[C@@H]1[C@H](C=C(O[C@H]1[C@@H]([C@@H](CO)O)O)C(=O)O)N2CCN(CC2)C3=CC=CC=C3 | doi:10.1016/j.ejmech.2012.06.033 |
| 453 | 893600 | Non-inhibitor | CC(=O)N[C@@H]1[C@H](C=C(O[C@H]1[C@@H]([C@@H](CO)O)O)C(=O)O)NC(=S)NC2=CC=C(C=C2)C(F)(F)F | doi:10.1016/j.ejmech.2012.06.033 |
| 454 | 1000000 | Non-inhibitor | CC(=O)N[C@@H]1[C@H](C=C(O[C@H]1[C@@H]([C@@H](CO)O)O)C(=O)O)NC(=S)NC2=CC=CC=C2Br | doi:10.1016/j.ejmech.2012.06.033 |
| 455 | 529700 | Non-inhibitor | CC(=O)N[C@@H]1[C@H](C=C(O[C@H]1[C@@H]([C@@H](CO)O)O)C(=O)O)NC(=O)NCC(=O)O | doi:10.1016/j.ejmech.2012.06.033 |
| 456 | 413200 | Non-inhibitor | CC(=O)N[C@@H]1[C@H](C=C(O[C@H]1[C@@H]([C@@H](CO)O)O)C(=O)O)NC(=O)N[C@@H](CCSC)C(=O)O | doi:10.1016/j.ejmech.2012.06.033 |
| 457 | 68500 | Non-inhibitor | CC(=O)N[C@@H]1[C@H](C=C(O[C@H]1[C@@H]([C@@H](CO)O)O)C(=O)O)NC(=O)NCC(=O)NCC(=O)O | doi:10.1016/j.ejmech.2012.06.033 |
| 458 | 57100 | Non-inhibitor | CC(=O)N[C@@H]1[C@H](C=C(O[C@H]1[C@@H]([C@@H](CO)O)O)C(=O)O)NC(=S)NC2=CC=CC=C2F | doi:10.1016/j.ejmech.2012.06.033 |
| 459 | 64500 | Non-inhibitor | CCCCNC(=S)N[C@H]1C=C(O[C@H]([C@@H]1NC(=O)C)[C@@H]([C@@H](CO)O)O)C(=O)O | doi:10.1016/j.ejmech.2012.06.033 |
| 460 | 549900 | Non-inhibitor | CC(=O)N[C@@H]1[C@H](C=C(O[C@H]1[C@@H]([C@@H](CO)O)O)C(=O)O)NC(=S)NC2CC2 | doi:10.1016/j.ejmech.2012.06.033 |
| 461 | 566800 | Non-inhibitor | CC(=O)N[C@@H]1[C@H](C=C(O[C@H]1[C@@H]([C@@H](CO)O)O)C(=O)O)N2CCOCC2 | doi:10.1016/j.ejmech.2012.06.033 |
| 462 | 271300 | Non-inhibitor | CC(=O)N[C@@H]1[C@H](C=C(O[C@H]1[C@@H]([C@@H](CO)O)O)C(=O)O)N2CCN(CC2)C3=CC=CC(=C3)C(F)(F)F | doi:10.1016/j.ejmech.2012.06.033 |
| 463 | 704700 | Non-inhibitor | CC(=O)N[C@@H]1[C@H](C=C(O[C@H]1[C@@H]([C@@H](CO)O)O)C(=O)O)NC(=O)NCC2=CC=CC=C2 | doi:10.1016/j.ejmech.2012.06.033 |
| 464 | 101300 | Non-inhibitor | C[C@@H](C(=O)NCC(=O)O)NC(=O)N[C@H]1C=C(O[C@H]([C@@H]1NC(=O)C)[C@@H]([C@@H](CO)O)O)C(=O)O | doi:10.1016/j.ejmech.2012.06.033 |
| 465 | 1000000 | Non-inhibitor | CC(=O)N[C@@H]1[C@H](C=C(O[C@H]1[C@@H]([C@@H](CO)O)O)C(=O)O)NC(=S)NC2=CC(=CC=C2)OC | doi:10.1016/j.ejmech.2012.06.033 |
| 466 | 674400 | Non-inhibitor | CC(=O)N[C@@H]1[C@H](C=C(O[C@H]1[C@@H]([C@@H](CO)O)O)C(=O)O)NC(=S)NC2=CC=CC3=CC=CC=C32 | doi:10.1016/j.ejmech.2012.06.033 |
| 467 | 611600 | Non-inhibitor | CCNC(=S)N[C@H]1C=C(O[C@H]([C@@H]1NC(=O)C)[C@@H]([C@@H](CO)O)O)C(=O)O | doi:10.1016/j.ejmech.2012.06.033 |
| 468 | 663000 | Non-inhibitor | C[C@@H](C(=O)O)NC(=O)N[C@H]1C=C(O[C@H]([C@@H]1NC(=O)C)[C@@H]([C@@H](CO)O)O)C(=O)O | doi:10.1016/j.ejmech.2012.06.033 |
| 469 | 54350 | Non-inhibitor | CC(C)[C@@H](C(=O)O)NC(=O)N[C@H]1C=C(O[C@H]([C@@H]1NC(=O)C)[C@@H]([C@@H](CO)O)O)C(=O)O | doi:10.1016/j.ejmech.2012.06.033 |
| 470 | 495600 | Non-inhibitor | CC(=O)N[C@@H]1[C@H](C=C(O[C@H]1[C@@H]([C@@H](CO)O)O)C(=O)O)N2CCCC(C2)C(=O)O | doi:10.1016/j.ejmech.2012.06.033 |
| 471 | 1000000 | Non-inhibitor | CC(=O)N[C@@H]1[C@H](C=C(O[C@H]1[C@@H]([C@@H](CO)O)O)C(=O)O)NC(=O)NC2=CC=C(C=C2)Cl | doi:10.1016/j.ejmech.2012.06.033 |
| 472 | 1000000 | Non-inhibitor | CC(=O)N[C@@H]1[C@H](C=C(O[C@H]1[C@@H]([C@@H](CO)O)O)C(=O)O)NC(=S)NC2=CC=CC(=C2)C(F)(F)F | doi:10.1016/j.ejmech.2012.06.033 |
| 473 | 487800 | Non-inhibitor | C[C@H]([C@@H](C(=O)O)NC(=O)N[C@H]1C=C(O[C@H]([C@@H]1NC(=O)C)[C@@H]([C@@H](CO)O)O)C(=O)O)O | doi:10.1016/j.ejmech.2012.06.033 |
| 474 | 580 | Inhibitor | CC(=O)N[C@@H]1[C@H](C=C(O[C@H]1[C@@H]([C@@H](CO)O)O)C(=O)O)NC(=O)N[C@@H](CC(=O)N)C(=O)O | doi:10.1016/j.ejmech.2012.06.033 |
| 475 | 58100 | Non-inhibitor | CC(=O)N[C@@H]1[C@H](C=C(O[C@H]1[C@@H]([C@@H](CO)O)O)C(=O)O)NC(=O)N[C@@H](CCC(=O)N)C(=O)O | doi:10.1016/j.ejmech.2012.06.033 |
| 476 | 510900 | Non-inhibitor | CC(=O)N[C@@H]1[C@H](C=C(O[C@H]1[C@@H]([C@@H](CO)O)O)C(=O)O)N2CCC3(CC2)OCCO3 | doi:10.1016/j.ejmech.2012.06.033 |
| 477 | 1000000 | Non-inhibitor | CC(=O)N[C@@H]1[C@H](C=C(O[C@H]1[C@@H]([C@@H](CO)O)O)C(=O)O)NC(=O)NC2=CC=C(C=C2)F | doi:10.1016/j.ejmech.2012.06.033 |
| 478 | 848100 | Non-inhibitor | CC(=O)N[C@@H]1[C@H](C=C(O[C@H]1[C@@H]([C@@H](CO)O)O)C(=O)O)NC(=O)NC2=CC=C(C=C2)OC | doi:10.1016/j.ejmech.2012.06.033 |
| 479 | 1000000 | Non-inhibitor | CC(=O)N[C@@H]1[C@H](C=C(O[C@H]1[C@@H]([C@@H](CO)O)O)C(=O)O)NC(=S)NC2=CC=CC=C2 | doi:10.1016/j.ejmech.2012.06.033 |
| 480 | 100000 | Non-inhibitor | CC(=O)N[C@@H]1[C@H](C=C(O[C@H]1[C@@H]([C@@H](CO)O)O)C(=O)O)NC(=S)NCCCl | doi:10.1016/j.ejmech.2012.06.033 |
| 481 | 573700 | Non-inhibitor | CC(=O)N[C@@H]1[C@H](C=C(O[C@H]1[C@@H]([C@@H](CO)O)O)C(=O)O)NC(=O)N2CCC[C@H]2C(=O)O | doi:10.1016/j.ejmech.2012.06.033 |
| 482 | 21 | Inhibitor | C1=C(O[C@H]([C@@H]([C@H]1N=C(N)N)NC(=O)C(F)(F)F)[C@@H]([C@@H](CO)O)O)C(=O)O | doi:10.1016/0223-5234(96)80447-8 |
| 483 | 86 | Inhibitor | CS(=O)(=O)N[C@@H]1[C@H](C=C(O[C@H]1[C@@H]([C@@H](CO)O)O)C(=O)O)N=C(N)N | doi:10.1016/0223-5234(96)80447-8 |
| 484 | 4300 | Inhibitor | CCC(=O)N[C@@H]1[C@H](C=C(O[C@H]1[C@@H]([C@@H](CO)O)O)C(=O)O)N | doi:10.1016/0223-5234(96)80447-8 |
| 485 | >430000 | Non-inhibitor | C1CC1C(=O)N[C@@H]2[C@H](C=C(O[C@H]2[C@@H]([C@@H](CO)O)O)C(=O)O)N | doi:10.1016/0223-5234(96)80447-8 |
| 486 | 540000 | Non-inhibitor | CC(=O)N(C)[C@@H]1[C@H](C=C(O[C@H]1[C@@H]([C@@H](CO)O)O)C(=O)O)N | doi:10.1016/0223-5234(96)80447-8 |
| 487 | >460000 | Non-inhibitor | C1CC(=O)N(C1)[C@@H]2[C@H](C=C(O[C@H]2[C@@H]([C@@H](CO)O)O)C(=O)O)N | doi:10.1016/0223-5234(96)80447-8 |
| 488 | 1700 | Inhibitor | CS(=O)(=O)N[C@@H]1[C@H](C=C(O[C@H]1[C@@H]([C@@H](CO)O)O)C(=O)O)N | doi:10.1016/0223-5234(96)80447-8 |
| 489 | 210000 | Non-inhibitor | CCS(=O)(=O)N[C@@H]1[C@H](C=C(O[C@H]1[C@@H]([C@@H](CO)O)O)C(=O)O)N | doi:10.1016/0223-5234(96)80447-8 |
| 490 | >340000 | Non-inhibitor | C1=C(O[C@H]([C@@H]([C@H]1N)NS(=O)(=O)C(F)(F)F)[C@@H]([C@@H](CO)O)O)C(=O)O | doi:10.1016/0223-5234(96)80447-8 |
| 491 | 320 | Inhibitor | [C@@H]1([C@@H]([C@H](C=C(O1)C(=O)O)N)NC(=O)C)[C@@H]([C@@H](CO)O)OC(=O)N | doi:10.1016/S0223-5234(00)80026-4 |
| 492 | 5 | Inhibitor | [C@@H]1([C@@H]([C@H](C=C(O1)C(=O)O)NC(N)=N)NC(=O)C)[C@@H]([C@@H](CO)O)OC(=O)N | doi:10.1016/S0223-5234(00)80026-4 |
| 493 | 6000 | Inhibitor | [C@@H]1([C@@H]([C@H](C=C(O1)C(=O)O)N)NC(=O)C)[C@@H]([C@@H](CO)O)OC(=O)NCCCCCC | doi:10.1016/S0223-5234(00)80026-4 |
| 494 | 3.7 | Inhibitor | [C@@H]1([C@@H]([C@H](C=C(O1)C(=O)O)NC(N)N)NC(=O)C)[C@@H]([C@@H](CO)O)OC(=O)NCCCCCCC | doi:10.1016/S0223-5234(00)80026-4 |
| 495 | 4200 | Inhibitor | CCCNC(=O)O[C@@H]([C@H]1[C@@H]([C@H](C=C(O1)C(=O)O)N)NC(=O)C)[C@@H](CO)O | doi:10.1016/S0223-5234(00)80026-4 |
| 496 | 9800 | Inhibitor | CCCN(CCC)C(=O)O[C@@H]([C@H]1[C@@H]([C@H](C=C(O1)C(=O)O)N)NC(=O)C)[C@@H](CO)O | doi:10.1016/S0223-5234(00)80026-4 |
| 497 | 76000 | Non-inhibitor | CCCCCCN(CCCCCC)C(=O)O[C@@H]([C@H]1[C@@H]([C@H](C=C(O1)C(=O)O)N)NC(=O)C)[C@@H](CO)O | doi:10.1016/S0223-5234(00)80026-4 |
| 498 | 100000 | Non-inhibitor | CC(=O)N[C@@H]1[C@H](C=C(O[C@H]1[C@@H]([C@@H](CO)O)OC(=O)N(C2CCCCC2)C3CCCCC3)C(=O)O)N | doi:10.1016/S0223-5234(00)80026-4 |
| 499 | 140 | Inhibitor | [C@@H]1([C@@H]([C@H](C=C(O1)C(=O)O)NC(N)=N)NC(=O)C)[C@@H]([C@@H](CO)O)OC(=O)NN2CCN(CC2)C(=O)OCC3=CC=CC=C3 | doi:10.1016/S0223-5234(00)80026-4 |
| 500 | 4 | Inhibitor | CC(=O)N[C@@H]1[C@H](C=C(O[C@H]1[C@@H]([C@@H](CO)O)OC(=O)NCCCCCCN)C(=O)O)N=C(N)N | doi:10.1016/S0223-5234(00)80026-4 |
| 501 | 720 | Inhibitor | CC(=O)N[C@@H]1[C@H](C=C(O[C@H]1[C@@H]([C@@H](CO)O)OC(=O)N(CCCCN)CCCN)C(=O)O)N=C(N)N | doi:10.1016/S0223-5234(00)80026-4 |
| 502 | 77 | Inhibitor | [C@@H]1([C@@H]([C@H](C=C(O1)C(=O)O)NC(N)=N)NC(=O)C)[C@@H]([C@@H](CO)O)OC(=O)NCCCCNCCN | doi:10.1016/S0223-5234(00)80026-4 |
| 503 | 38 | Inhibitor | [C@@H]1([C@@H]([C@H](C=C(O1)C(=O)O)NC(N)=N)NC(=O)C)[C@@H]([C@@H](CO)O)OC(=O)NN2CCNCC2 | doi:10.1016/S0223-5234(00)80026-4 |
| 504 | 6.2 | Inhibitor | CC(=O)N[C@@H]1[C@H](C=C(O[C@H]1[C@@H]([C@@H](CO)O)OC(=O)NCCSC2=NC=CC=N2)C(=O)O)N=C(N)N | doi:10.1016/S0223-5234(00)80026-4 |
| 505 | 13 | Inhibitor | [C@@H]1([C@@H]([C@H](C=C(O1)C(=O)O)NC(N)=N)NC(=O)C)[C@@H]([C@@H](CO)O)OC(=O)NCCO[C@H]2[C@@H]([C@@H]([C@H]([C@@H](O2)CO)O)O)O | doi:10.1016/S0223-5234(00)80026-4 |
| 506 | 0.743 | Inhibitor | N[C@@H]1[C@H]([C@@H](C=C(C1)C(=O)O)OC(CC)CC)NC(CF)=O | doi:10.1016/j.antiviral.2013.10.008 |
| 507 | 0.598 | Inhibitor | N[C@@H]1[C@H]([C@@H](C=C(C1)C(=O)O)OC(CC)CC)NC(C(F)F)=O | doi:10.1016/j.antiviral.2013.10.008 |
| 508 | 0.743 | Inhibitor | N[C@@H]1[C@H]([C@@H](C=C(C1)C(=O)O)OC(CC)CC)NC(C(F)(F)F)=O | doi:10.1016/j.antiviral.2013.10.008 |
| 509 | 4700 | Inhibitor | CC1=CC=C(C=C1)C2=CN(C(=N2)N)C3=C(C=CC(=C3)C(=O)O)NC(=O)C | doi:10.1016/S0223-5234(99)80055-5 |
| 510 | 150000 | Non-inhibitor | CC(=O)NC1=C(C=C(C=C1)C(=O)O)N2C=C(N=C2N)C3=CC(=CC=C3)[N+](=O)[O-] | doi:10.1016/S0223-5234(99)80055-5 |
| 511 | 5300 | Inhibitor | CC(=O)NC1=C(C=C(C=C1)C(=O)O)N2C=C(N=C2N)C3=CC=CO3 | doi:10.1016/S0223-5234(99)80055-5 |
| 512 | 210000 | Non-inhibitor | CC(=O)NC1=C(C=C(C=C1)C(=O)O)N2C=C(N=C2N)C3=CC(=CO3)C(=O)OC | doi:10.1016/S0223-5234(99)80055-5 |
| 513 | 98000 | Non-inhibitor | CC(=O)NC1=C(C=C(C=C1)C(=O)O)N2C=C(N=C2N)C3=COC4=CC=CC=C43 | doi:10.1016/S0223-5234(99)80055-5 |
| 514 | 9400 | Inhibitor | CCC1=CN(C(=N1)N)C2=C(C=CC(=C2)C(=O)O)NC(=O)C | doi:10.1016/S0223-5234(99)80055-5 |
| 515 | 7000 | Inhibitor | CC(=O)NC1=C(C=C(C=C1)C(=O)O)N2C=CN=C2N | doi:10.1016/S0223-5234(99)80055-5 |
| 516 | >510000 | Non-inhibitor | CC(=O)NC1=C(C=C(C=C1)C(=O)O)N2C=CN=C2 | doi:10.1016/S0223-5234(99)80055-5 |
| 517 | >560000 | Non-inhibitor | CCC1=NC=CN1C2=C(C=CC(=C2)C(=O)O)NC(=O)C | doi:10.1016/S0223-5234(99)80055-5 |
| 518 | >450000 | Non-inhibitor | C1=CC(=C(C=C1C(=O)O)N2C=CN=C2N)N | doi:10.1016/S0223-5234(99)80055-5 |
| 519 | 770 | Inhibitor | CCC(CC)O[C@H]1[C@@H]([C@H](C=C(O1)C(=O)O)N)NC(=O)C | doi:10.1016/S0960-894X(99)00031-1 |
| 520 | 39 | Inhibitor | CCC(CC)C([C@H]1[C@@H]([C@H](C=C(O1)C(=O)O)N)NC(=O)C)O | doi:10.1016/S0960-894X(99)00031-1 |
| 521 | 2 | Inhibitor | CCC(CC)C(=O)[C@H]1[C@@H]([C@H](C=C(O1)C(=O)O)N)NC(=O)C | doi:10.1016/S0960-894X(99)00031-1 |
| 522 | >660000 | Non-inhibitor | CCCO[C@@H]1C[C@]2(CC[C@]1([C@H](C2)O)NC(=O)C)C(=O)O | doi:10.1016/S0960-894X(99)00033-5 |
| 523 | 210 | Inhibitor | C(C)(=O)N[C@@H]1CC=C(C[C@H]1C(N(CC)CC)=O)C(=O)O | doi:10.1016/S0040-4039(01)00798-5 |
| 524 | 17 | Inhibitor | C(C)(=O)N[C@H]1[C@@H](C=C(CC1)C(=O)O)C(N(CC)CC)=O | doi:10.1016/S0040-4039(01)00798-5 |
| 525 | 1500 | Inhibitor | C(C)(=O)N[C@H]1[C@@H](C=C(OC1)C(=O)O)C(N(CC)CC)=O | doi:10.1016/S0040-4039(01)01887-1 |
| 526 | 71 | Inhibitor | C(C)(=O)N[C@H]1[C@@H](C=C(CC1)C(=O)O)OC(CC)CC | doi:10.1016/S0040-4039(01)01605-7 |
| 527 | 172000 | Non-inhibitor | C(C)(=O)N[C@H]1[C@H](C=C(CC1)C(=O)O)OC(CC)CC | doi:10.1016/S0040-4039(01)01605-7 |
| 528 | 4000000 | Non-inhibitor | CC(=O)NC1=C(C=C(C=C1)C(=O)O)NC(=O)CO | doi:10.1021/jm00017a005 |
| 529 | 10000000 | Non-inhibitor | CC(=O)NC1=C(C=C(C=C1)C(=O)O)OC(=O)C | doi:10.1021/jm00017a005 |
| 530 | 10000000 | Non-inhibitor | CC(=O)NC1=C(C=C(C=C1O)C(=O)O)N | doi:10.1021/jm00017a005 |
| 531 | 10000000 | Non-inhibitor | CC(=O)NC1=C(C=C(C=C1)C(=O)O)NC(=O)CCCN | doi:10.1021/jm00017a005 |
| 532 | 5000000 | Non-inhibitor | CC(=O)NC1=C(C=C(C=C1)C(=O)O)NC(=O)CN | doi:10.1021/jm00017a005 |
| 533 | 5000000 | Non-inhibitor | CC(=O)NC1=C(C=C(C=C1OC(=O)C)C(=O)O)[N+](=O)[O-] | doi:10.1021/jm00017a005 |
| 534 | 1000000 | Non-inhibitor | CC(=O)NC1=CC(=C(C(=C1O)[N+](=O)[O-])C(=O)O)[N+](=O)[O-] | doi:10.1021/jm00017a005 |
| 535 | 750000 | Non-inhibitor | CC(=O)NC1=C(C=C(C=C1O)C(=O)O)[N+](=O)[O-] | doi:10.1021/jm00017a005 |
| 536 | 5000000 | Non-inhibitor | CC(=O)NC1=C(C=C(C=C1)C(=O)O)NC(=O)C(CO)O | doi:10.1021/jm00017a005 |
| 537 | 8 | Inhibitor | O1C([C@@H]([C@H](O)CO)O)[C@H](NC(O)=O)[C@@H](NC(=N)NC(CN2N=NC(C3=CC=CC=C3)=C2)=O)C=C1C(=O)O | doi:10.1016/j.ejmech.2016.07.064 |
| 538 | 123 | Inhibitor | O1C([C@@H]([C@H](O)CO)O)[C@H](NC(O)=O)[C@@H](NC(=N)NC(CCN2N=NC(C3=CC=CC=C3)=C2)=O)C=C1C(=O)O | doi:10.1016/j.ejmech.2016.07.064 |
| 539 | 228 | Inhibitor | O1C([C@@H]([C@H](O)CO)O)[C@H](NC(O)=O)[C@@H](NC(=N)NC(CCCN2N=NC(C3=CC=CC=C3)=C2)=O)C=C1C(=O)O | doi:10.1016/j.ejmech.2016.07.064 |
| 540 | 110 | Inhibitor | O1C([C@@H]([C@H](O)CO)O)[C@H](NC(O)=O)[C@@H](NC(=N)NC(CCCCN2N=NC(C3=CC=CC=C3)=C2)=O)C=C1C(=O)O | doi:10.1016/j.ejmech.2016.07.064 |
| 541 | 2.9 | Inhibitor | O1C([C@@H]([C@H](O)CO)O)[C@H](NC(=O)O)[C@@H](NC(=N)NC(CN2N=NC(C3CCCCC3)=C2)=O)C=C1C(=O)O | doi:10.1016/j.ejmech.2016.07.064 |
| 542 | 17 | Inhibitor | O1C([C@@H]([C@H](O)CO)O)[C@H](NC(=O)O)[C@@H](NC(=N)NC(CN2N=NC(C3CCCCC=3)=C2)=O)C=C1C(=O)O | doi:10.1016/j.ejmech.2016.07.064 |
| 543 | 10 | Inhibitor | O1C([C@@H]([C@H](O)CO)O)[C@H](NC(=O)O)[C@@H](NC(=N)NC(CN2N=NC(C3=CC=CC=C3OC)=C2)=O)C=C1C(=O)O | doi:10.1016/j.ejmech.2016.07.064 |
| 544 | 13 | Inhibitor | O1C([C@@H]([C@H](O)CO)O)[C@H](NC(=O)O)[C@@H](NC(=N)NC(CN2N=NC(C3=CC=CC=C3N)=C2)=O)C=C1C(=O)O | doi:10.1016/j.ejmech.2016.07.064 |
| 545 | 8 | Inhibitor | O1C([C@@H]([C@H](O)CO)O)[C@H](NC(=O)O)[C@@H](NC(=N)NC(CN2N=NC(C3=CC=CC(Cl)=C3)=C2)=O)C=C1C(=O)O | doi:10.1016/j.ejmech.2016.07.064 |
| 546 | 17 | Inhibitor | O1C([C@@H]([C@H](O)CO)O)[C@H](NC(=O)O)[C@@H](NC(=N)NC(CN2N=NC(C3=CC=CC(F)=C3)=C2)=O)C=C1C(=O)O | doi:10.1016/j.ejmech.2016.07.064 |
| 547 | 11 | Inhibitor | O1C([C@@H]([C@H](O)CO)O)[C@H](NC(=O)O)[C@@H](NC(=N)NC(CN2N=NC(C3=CC=CC(N)=C3)=C2)=O)C=C1C(=O)O | doi:10.1016/j.ejmech.2016.07.064 |
| 548 | 19 | Inhibitor | O1C([C@@H]([C@H](O)CO)O)[C@H](NC(=O)O)[C@@H](NC(=N)NC(CN2N=NC(C3=CC=CC(C(=O)OCC)=C3)=C2)=O)C=C1C(=O)O | doi:10.1016/j.ejmech.2016.07.064 |
| 549 | 17 | Inhibitor | O1C([C@@H]([C@H](O)CO)O)[C@H](NC(=O)O)[C@@H](NC(=N)NC(CN2N=NC(C3=CC=C(OC)C=C3)=C2)=O)C=C1C(=O)O | doi:10.1016/j.ejmech.2016.07.064 |
| 550 | 13 | Inhibitor | O1C([C@@H]([C@H](O)CO)O)[C@H](NC(=O)O)[C@@H](NC(=N)NC(CN2N=NC(C3=CC=C(N)C=C3)=C2)=O)C=C1C(=O)O | doi:10.1016/j.ejmech.2016.07.064 |
| 551 | 17 | Inhibitor | O1C([C@@H]([C@H](O)CO)O)[C@H](NC(=O)O)[C@@H](NC(=N)NC(CN2N=NC(C3=CC=C(C)C=C3)=C2)=O)C=C1C(=O)O | doi:10.1016/j.ejmech.2016.07.064 |
| 552 | 14 | Inhibitor | O1C([C@@H]([C@H](O)CO)O)[C@H](NC(=O)O)[C@@H](NC(=N)NC(CN2N=NC(C3=CC=C(Cl)C=C3)=C2)=O)C=C1C(=O)O | doi:10.1016/j.ejmech.2016.07.064 |
| 553 | 27 | Inhibitor | O1C([C@@H]([C@H](O)CO)O)[C@H](NC(=O)O)[C@@H](NC(=N)NC(CN2N=NC(C3=CC=C(F)C=C3)=C2)=O)C=C1C(=O)O | doi:10.1016/j.ejmech.2016.07.064 |
| 554 | 28 | Inhibitor | O1C([C@@H]([C@H](O)CO)O)[C@H](NC(=O)O)[C@@H](NC(=N)NC(CN2N=NC(C3=CC4C=CC(OC)=CC=4C=C3)=C2)=O)C=C1C(=O)O | doi:10.1016/j.ejmech.2016.07.064 |
| 555 | 1.9 | Inhibitor | C(C)(=O)N[C@@H](C(CC)CC)[C@@H]1[C@@H]([C@H](C[C@H]1NC(=N)N)P(O)(O)=O)O | doi:10.1021/acs.jmedchem.6b00029 |
| 556 | 2.3 | Inhibitor | C(C)(=O)N[C@@H](C(CC)CC)[C@@H]1C=C(C[C@H]1NC(=N)N)P(O)(O)=O | doi:10.1021/acs.jmedchem.6b00029 |
| 557 | 6.4 | Inhibitor | C(C)(=O)N[C@@H](C(CC)CC)[C@@H]1C=C(C[C@H]1NC(=N)N)P([O-])([O-])=O | doi:10.1021/acs.jmedchem.6b00029 |
| 558 | 61000 | Non-inhibitor | C1=CC(=CC=C1C2=C(C(=O)C3=C(C=C(C=C3O2)O)O)O[C@H]4[C@@H]([C@H]([C@@H]([C@H](O4)CO)O)O)O[C@H]5[C@@H]([C@H]([C@@H]([C@H](O5)CO)O)O)O)O | doi:10.1055/s-0041-111631 |
| 559 | 51600 | Non-inhibitor | C[C@@H]1[C@H]([C@@H]([C@@H]([C@H](O1)O[C@@H]2[C@H]([C@@H]([C@H](O[C@H]2OC3=C(OC4=CC(=CC(=C4C3=O)O)O)C5=CC=C(C=C5)O)CO)O)O)O)O)O | doi:10.1055/s-0041-111631 |
| 560 | 52200 | Non-inhibitor | C1[C@H](C([C@H]([C@@H](O1)OC2[C@@H](OC([C@H](C2O)O)CO)OC3=C(OC4=CC(=CC(=C4C3=O)O)O[C@H]5C([C@H]([C@@H](C(O5)CO)O)O)O)C6=CC=C(C=C6)O)O)O)O | doi:10.1055/s-0041-111631 |
| 561 | 54000 | Non-inhibitor | C1=CC(=CC=C1C2=C(C(=O)C3=C(O2)C=C(C=C3[O-])O)O[C@H]4[C@@H]([C@H]([C@@H]([C@H](O4)CO)O)O)O)O | doi:10.1055/s-0041-111631 |
| 562 | 112800 | Non-inhibitor | C1=CC(=C(C=C1C2=C(C(=O)C3=C(C=C(C=C3O2)O)O)O[C@H]4C(C([C@@H](C(O4)CO)O)O)O[C@H]5[C@@H](C([C@@H](C(O5)CO)O)O)O)O)O | doi:10.1055/s-0041-111631 |
| 563 | 143200 | Non-inhibitor | COC1=C(C=C2C3CC4=C(CN3CCC2=C1)C5=C(C=C4)OCO5)O | doi:10.1055/s-0041-111631 |
| 564 | 88040 | Non-inhibitor | CC1[C@@H]([C@@H](C([C@@H](O1)OCC2[C@@H](C(C([C@@H](O2)OC3=C(OC4=CC(=CC(=C4C3=O)O)O)C5=CC=C(C=C5)O)O)O)O)O)O)O | doi:10.1016/j.phytochem.2016.06.011 |
| 565 | 84520 | Non-inhibitor | CC1[C@@H]([C@@H](C([C@@H](O1)OCC2[C@@H](C(C([C@@H](O2)OC3=C(OC4=CC(=CC(=C4C3=O)O)O)C5=CC(=C(C=C5)OC)O)O)O)O)O)O)O | doi:10.1016/j.phytochem.2016.06.011 |
| 566 | 137000 | Non-inhibitor | OC=1C=C(C=CC1O)/C=C/C(=O)NC1=CC(=C(C=C1)O)NC(CC1=CC=CC=C1)=O | doi:10.1007/s40242-015-5356-z |
| 567 | 166000 | Non-inhibitor | OC1=C(C=C(C=C1)NC(\C=C\C1=CC(=C(C=C1)O)OC)=O)NC(CC1=CC=CC=C1)=O | doi:10.1007/s40242-015-5356-z |
| 568 | 162000 | Non-inhibitor | O1C(=CC=C1)/C=C/C(=O)NC1=CC(=C(C=C1)O)NC(CC1=CC=CC=C1)=O | doi:10.1007/s40242-015-5356-z |
| 569 | 51500 | Non-inhibitor | OC=1C=C(C=CC1O)/C=C/C(=O)NC=1C=CC(=C(C1)NC(C1=C(C=CC=C1)O)=O)O | doi:10.1007/s40242-015-5356-z |
| 570 | 142000 | Non-inhibitor | C(C)(=O)NC=1C=C(C=CC1O)NC(\C=C\C=1OC=CC1)=O | doi:10.1007/s40242-015-5356-z |
| 571 | 147000 | Non-inhibitor | C(C)C(C(=O)NC1=C(C=CC(=C1)NC(\C=C\C=1OC=CC1)=O)O)CC | doi:10.1007/s40242-015-5356-z |
| 572 | 136000 | Non-inhibitor | OC1=C(C(=O)NC2=C(C=CC(=C2)NC(\C=C\C=2OC=CC2)=O)O)C=CC=C1 | doi:10.1007/s40242-015-5356-z |
| 573 | >200000 | Non-inhibitor | C[C@H](CC/C=C(\C)/C(=O)O)[C@H]1C[C@@H]([C@@]2([C@@]1(CC=C3C2=CC[C@@H]4[C@@]3(CCC(=O)C4(C)C)C)C)C)OC(=O)C | doi:10.1038/srep13194 |
| 574 | >200000 | Non-inhibitor | C[C@H](CC/C=C(\C)/C(=O)O)[C@H]1CC([C@@]2([C@@]1(CC=C3C2=CC[C@@H]4[C@@]3(CCC(=O)C4(C)C)C)C)C)O | doi:10.1038/srep13194 |
| 575 | >200000 | Non-inhibitor | C[C@H](CC/C=C(\C)/C(=O)O)[C@H]1C[C@@H]([C@@]2([C@@]1(CC=C3C2=CCC4[C@@]3(CC[C@@H](C4(C)C)O)C)C)C)OC(=O)C | doi:10.1038/srep13194 |
| 576 | >200000 | Non-inhibitor | C[C@H](CC/C=C(/C)\C(=O)O)[C@H]1CC[C@@]2([C@@]1(CC=C3C2=CCC4[C@@]3(CCC(=O)C4(C)C)C)C)C | doi:10.1038/srep13194 |
| 577 | >200000 | Non-inhibitor | C[C@H](CC/C=C(\C)/C(=O)O)[C@H]1CC[C@@]2([C@@]1(CC=C3C2=CC[C@@H]4[C@@]3(CCC(=O)C4(C)C)C)C)C | doi:10.1038/srep13194 |
| 578 | >200000 | Non-inhibitor | C[C@H](CC/C=C(\C)/C(=O)O)[C@H]1CC[C@@]2([C@@]1(CC=C3C2=CC[C@@H]4[C@@]3(CC[C@@H](C4(C)C)O)C)C)C | doi:10.1038/srep13194 |
| 579 | >200000 | Non-inhibitor | C[C@H](CC(=O)C[C@@H](C)C(=O)O)[C@H]1C[C@@H]([C@@]2([C@@]1(CC(=O)C3=C2[C@H](C[C@@H]4[C@@]3(CCC(=O)C4(C)C)C)O)C)C)O | doi:10.1038/srep13194 |
| 580 | >200000 | Non-inhibitor | C[C@H](CC(=O)/C=C(\C)/[C@H]1C[C@@H]([C@@]2([C@@]1(CC(=O)C3=C2[C@H](C[C@@H]4[C@@]3(CCC(=O)C4(C)C)C)O)C)C)O)C(=O)O | doi:10.1038/srep13194 |
| 581 | >200000 | Non-inhibitor | C[C@H](CC(=O)C[C@@H](C)C(=O)O)[C@H]1C[C@@H]([C@@]2([C@@]1(CC(=O)C3=C2[C@H](C[C@@H]4[C@@]3(CC[C@@H](C4(C)C)O)C)O)C)C)O | doi:10.1038/srep13194 |
| 582 | >200000 | Non-inhibitor | C[C@H](CC(=O)CC(C)C(=O)O)[C@H]1CC(=O)[C@@]2([C@@]1(CC(=O)C3=C2C(=O)C[C@@H]4[C@@]3(CC[C@@H](C4(C)C)O)C)C)C | doi:10.1038/srep13194 |
| 583 | >200000 | Non-inhibitor | C[C@H](CC(=O)CC(C)C(=O)O)[C@H]1CC(=O)[C@@]2([C@@]1([C@@H](C(=O)C3=C2[C@H](C[C@@H]4[C@@]3(CC[C@@H](C4(C)C)O)C)O)OC(=O)C)C)C | doi:10.1038/srep13194 |
| 584 | >200000 | Non-inhibitor | C[C@H](CC(=O)/C=C(/C)\[C@H]1CC(=O)[C@@]2([C@@]1(CC(=O)C3=C2C(=O)CC4[C@@]3(CC[C@@H](C4(C)C)O)C)C)C)C(=O)O | doi:10.1038/srep13194 |
| 585 | >200000 | Non-inhibitor | C[C@@H](CC(=O)C[C@@H](C)C(=O)O)[C@H]1CC(=O)[C@@]2([C@@]1([C@@H](C(=O)C3=C2C(=O)C[C@@H]4[C@@]3(CC[C@@H](C4(C)C)O)C)OC(=O)C)C)C | doi:10.1038/srep13194 |
| 586 | >200000 | Non-inhibitor | C[C@H](CC(=O)C[C@@H](C)C(=O)O)[C@H]1CC(=O)[C@@]2([C@@]1(CC(=O)C3=C2[C@H](C[C@@H]4[C@@]3(CC[C@@H](C4(C)C)O)C)O)C)C | doi:10.1038/srep13194 |
| 587 | >200000 | Non-inhibitor | C[C@H](CC(=O)/C=C(/C)\[C@H]1CC(=O)[C@@]2([C@@]1(CC(=O)C3=C2C(=O)CC4[C@@]3(CCC(=O)C4(C)C)C)C)C)C(=O)O | doi:10.1038/srep13194 |
| 588 | >200000 | Non-inhibitor | C[C@H](CC(=O)/C=C(/C)\[C@H]1C[C@@H]([C@@]2([C@@]1(CC(=O)C3=C2[C@H](CC4[C@@]3(CC[C@@H](C4(C)C)O)C)O)C)C)O)C(=O)O | doi:10.1038/srep13194 |
| 589 | >200000 | Non-inhibitor | CC(CC(=O)/C=C(/C)\C1CC(=O)[C@@]2([C@@]1(CC(=O)C3=C2[C@H](CC4[C@@]3(CCC(=O)C4(C)C)C)O)C)C)C(=O)O | doi:10.1038/srep13194 |
| 590 | >200000 | Non-inhibitor | C[C@H](CC(=O)C[C@@H](C)C(=O)O)[C@H]1CC(=O)[C@@]2([C@@]1([C@@H](C(=O)C3=C2C(=O)C[C@@H]4[C@@]3(CC[C@@H](C4(C)C)O)C)O)C)C | doi:10.1038/srep13194 |
| 591 | >200000 | Non-inhibitor | C[C@H](CC(=O)C[C@@H](C)C(=O)O)[C@H]1CC(=O)[C@@]2([C@@]1(CC(=O)C3=C2[C@H](C[C@@H]4[C@@]3(CCC(=O)C4(C)C)C)O)C)C | doi:10.1038/srep13194 |
| 592 | >200000 | Non-inhibitor | C[C@H](CC/C=C(\C)/C(=O)O)[C@H]1CC[C@@]2([C@@]1(CCC3=C2C(=O)C[C@@H]4[C@@]3(CCC(=O)C4(C)C)C)C)C | doi:10.1038/srep13194 |
| 593 | >200000 | Non-inhibitor | C[C@H](CC(=O)C[C@@H](C)C(=O)O)[C@H]1C[C@@H]([C@@]2([C@@]1(CC(=O)C3=C2CC[C@@H]4[C@@]3(CCC(=O)C4(C)C)C)C)C)O | doi:10.1038/srep13194 |
| 594 | >200000 | Non-inhibitor | C[C@H](C[C@@H](/C=C(\C)/C(=O)O)O)[C@H]1CC(=O)[C@@]2([C@@]1(CC(=O)C3=C2C(=O)C[C@@H]4[C@@]3(CC[C@@H](C4(C)C)O)C)C)C | doi:10.1038/srep13194 |
| 595 | >200000 | Non-inhibitor | C[C@H](C[C@@H](/C=C(\C)/C(=O)O)O)[C@H]1CC(=O)[C@@]2([C@@]1(CC(=O)C3=C2[C@H](CC4[C@@]3(CCC(=O)C4(C)C)C)O)C)C | doi:10.1038/srep13194 |
| 596 | >200000 | Non-inhibitor | C[C@H](CC(=O)CC(C)C(=O)O)[C@H]1CC(=O)[C@@]2([C@@]1([C@@H](C(=O)C3=C2C(=O)C[C@@H]4[C@@]3(CCC(=O)C4(C)C)C)OC(=O)C)C)C | doi:10.1038/srep13194 |
| 597 | >200000 | Non-inhibitor | C[C@H](CC/C=C(\C)/CO)[C@H]1CC[C@@]2([C@@]1(CC=C3C2=CC[C@@H]4[C@@]3(CCC(=O)C4(C)C)C)C)C | doi:10.1038/srep13194 |
| 598 | >200000 | Non-inhibitor | C[C@H](CC/C=C(\C)/CO)[C@H]1CC[C@@]2([C@@]1(CC=C3C2=CC[C@@H]4[C@@]3(CC[C@@H](C4(C)C)O)C)C)C | doi:10.1038/srep13194 |
| 599 | >200000 | Non-inhibitor | C[C@H](CCC=C(CO)CO)[C@H]1CC[C@@]2([C@@]1(CC=C3C2=CC[C@@H]4[C@@]3(CCC(=O)C4(C)C)C)C)C | doi:10.1038/srep13194 |
| 600 | >200000 | Non-inhibitor | C[C@H](CC[C@@H](C(C)(C)O)O)[C@H]1CC[C@@]2([C@@]1(CC=C3C2=CC[C@@H]4[C@@]3(CCC(=O)C4(C)C)C)C)C | doi:10.1038/srep13194 |
| 601 | >200000 | Non-inhibitor | CC(CCC(C(C)(CO)O)O)C1CCC2(C1(CC=C3C2=CCC4C3(CCC(=O)C4(C)C)C)C)C | doi:10.1038/srep13194 |
| 602 | >200000 | Non-inhibitor | C[C@H](CC/C=C(\C)/C=O)[C@H]1CC[C@@]2([C@@]1(CC=C3C2=CCC4[C@@]3(CC[C@@H](C4(C)C)O)C)C)C | doi:10.1038/srep13194 |
| 603 | >200000 | Non-inhibitor | C[C@H](CC/C=C(\C)/C=O)[C@H]1CC[C@@]2([C@@]1(CCC3=C2C(=O)C[C@@H]4[C@@]3(CCC(=O)C4(C)C)C)C)C | doi:10.1038/srep13194 |
